# Multivalent polyglycerol supported imidazolidin-4-one organocatalysts for enantioselective Friedel–Crafts alkylations

**DOI:** 10.3762/bjoc.11.83

**Published:** 2015-05-12

**Authors:** Tommaso Pecchioli, Manoj Kumar Muthyala, Rainer Haag, Mathias Christmann

**Affiliations:** 1 Institut für Chemie und Biochemie, Freie Universität Berlin, Takustraße 3, 14195 Berlin, Germany

**Keywords:** Friedel–Crafts, homogeneous catalysis, hyperbranched polyglycerol, imidazolidin-4-one, multivalency

## Abstract

The first immobilization of a MacMillan’s first generation organocatalyst onto dendritic support is described. A modified tyrosine-based imidazolidin-4-one was grafted to a soluble high-loading hyperbranched polyglycerol via a copper-catalyzed alkyne–azide cycloaddition (CuAAC) reaction and readily purified by dialysis. The efficiency of differently functionalized multivalent organocatalysts **4a–c** was tested in the asymmetric Friedel–Crafts alkylation of *N*-methylpyrrole with α,β-unsaturated aldehydes. A variety of substituted enals was investigated to explore the activity of the catalytic system which was also compared with monovalent analogues. The catalyst **4b** showed excellent turnover rates and no loss of activity due to immobilization, albeit moderate enantioselectivities were observed. Moreover, easy recovery by selective precipitation allowed the reuse of the catalyst for three cycles.

## Introduction

In nature, multivalent architectures, e.g., enzymes, bacteria or viruses, are responsible for cooperative interactions between different interfaces or molecules [1]. The realization of the concept of multivalency has attracted attention from different fields ranging from medicine and biochemistry [2] to supramolecular chemistry [3–4] and materials sciences [5]. However, applications in catalysis are still limited [6–8]. Recently, the use of polymeric support has stimulated the development of multivalent architectures for catalytic applications [9]. In general, both linear and various families of branched polymers such as dendrimers, dendritic-hybrid and hyperbranched polymers are used as macromolecular support for catalysis [10–12]. Linear polymers such as poly(ethylene glycol) (PEG) [13] or non-cross-linked polystyrene (NCPS) [14] are readily available but suffer from poor loading capacity, while in the case of dendrimers, the highest loading can be achieved due to their extraordinary branching [15]. These well-defined molecules are soluble in many organic solvents and can combine the advantages of hetero- and homogeneous catalysis [16–17]. However, their tedious and multistep syntheses using either divergent or convergent approaches are arguably the reason for their limited use as support in organic synthesis [18]. To overcome these obstacles, a hybrid dendron-polymer might constitute a valuable alternative for high-loading platforms [19], despite the use of solid support such as polystyrene may lead to the disadvantage of operating in heterogeneous media. In contrast to the stepwise syntheses of dendrimers and dendron hybrids, the hyperbranched polymers can be easily obtained in kilogram scale through one-pot reactions [10], maintaining properties such as high loading capacity combined with the solubility characteristics of the respective dendrimers [20–21]. As a macromolecule, the supported catalyst can be recovered from the reaction media by selective precipitation, dialysis or filtration techniques, depending on its particular physical properties. Hyperbranched polymers like polytriallylsilane or polyglycerol have been used in a wide range of transformations including aldol condensations [22], Suzuki cross-couplings [23] and Diels–Alder reactions [24], to name a few, with metal complexes as catalytically active principle.

The advent of organocatalysis has allowed for selective C–C bond formation by using small organic molecules [25–31]. In contrast to metal complexes, chiral or achiral organocatalysts are easily attached on supports. They do not suffer from metal leaching and they can be reused more readily [32–36]. Moreover, their stability allows to perform reactions under mild and aerobic conditions and in the presence of water, both as co-solvent or the only solvent [37]. In the last years, several reports on water effects in organocatalytic reactions were published [38–42]. The use of supported catalyst has proven beneficial with regard to rate acceleration and increased selectivity due to formation of an aqueous microenvironment favored by the swelling properties of polymeric materials [43]. Particularly, in the case of dendritic proline derivatives [44–46] and *N*-alkylimidazole decorated dendron-hybrids [47], the presence of water was crucial for aldol and Baylis–Hillman reactions, as recently reported by Miller and Portnoy [48].

To the best of our knowledge, the immobilization of chiral organocatalysts on hyperbranched polymeric support has remained unexplored. Therefore, we decided to use hyperbranched polyglycerol (hPG) [49] as a polymeric support. The high local concentration of hydrophilic functionality present on its periphery is especially attractive since it might promote water coordination. These properties prompted us to investigate the effects of high-loading support in asymmetric organocatalysis.

The use of chiral imidazolidinones in organocatalysis has been extensively reported for a wide range of enantioselective reactions involving α,β-unsaturated aldehydes, such as the Diels–Alder reactions [50], 1,3-dipolar cycloadditions [51] and Friedel–Crafts alkylations [52–53]. To date, heterogenizations have been applied mainly in Diels–Alder reactions [54–61]. Nevertheless, Friedel–Crafts alkylations are recently emerging as a compelling field of study as reported by Pericàs [62] and others [58,60]. The simple approach providing an enantioselective entry to new C–C bonds allows for the use of readily available starting materials and can typically be carried out in THF–water mixtures. Our aim was to employ this transformation as a benchmark in order to explore the efficiency of novel multivalent architectures.

Herein, we describe the first immobilization of imidazolidin-4-one onto hyperbranched polyglycerol (hPG) and its application as multivalent organocatalyst.

## Results and Discussion

**Scheme 1:**
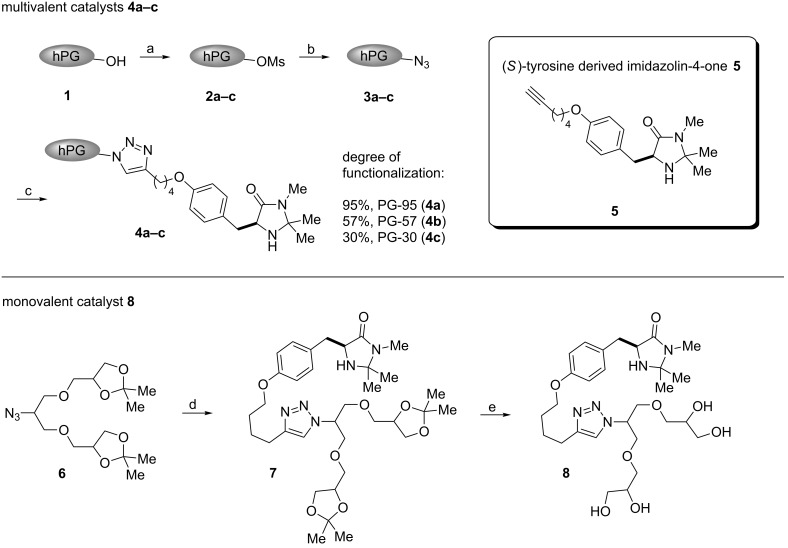
Synthesis of hyperbranched polyglycerol-supported and G1 dendronized imidazolidin-4-ones **4a–c** and **8** using a CuAAC reaction. Reaction conditions: (a) **1** (1.0 equiv), MsCl (1.2 equiv, with respect to degrees of functionalization), pyridine, 25 °C, 16 h, 76% **2a**, 82% **2b** and 87% **2c**. (b) **2a–c** (1.0 equiv), NaN_3_ (3.0 equiv), DMF, 65 °C, 72 h, 72% **3a**, 81% **3b** and 86% **3c**. (c) **3a–c** (1.0 equiv), **5** (2.0 equiv), CuSO_4_·5H_2_O (0.2 equiv), sodium ascorbate (2.0 equiv), THF/H_2_O 3:1 (v/v), 25 °C, 48 h, 71% **4a**, 40% **4b** and 35% **4c**. (d) **6** (1.1 equiv), **5** (1.0 equiv), CuSO_4_·5H_2_O (0.1 equiv), sodium ascorbate (0.2 equiv), DIPEA (0.1 equiv), THF/H_2_O 3:1 (v/v), 25 °C, 12 h, 70%. (e) **7**, Dowex 50, MeOH, reflux, 12 h, 95%.

To explore the synthetic utility of hPG in organocatalysis, we here report the synthesis and application of a series of three multivalent dendronized imidazolidin-4-ones PG-95 (**4a**), PG-57 (**4b**) and PG-30 (**4c**) representing different degrees of functionalization: 95% (**4a**), 57% (**4b**) and 30% (**4c**), respectively. An (*S*)-tyrosine-derived imidazolidin-4-one **5** was anchored to the polymeric support through a CuAAC reaction. Following the same strategy, a monovalent analog **8** bearing a G1 glycerol dendron tail was also prepared for comparison with the multivalent systems **4a–c** and evaluation of the possible presence of cooperative effects (Scheme 1).

Polyglycerol **1** (*M*_n_ = 9000 g/mol, loading OH = 13.5 mmol/g, PDI = 1.87) was obtained following a previously reported procedure by a one-step ring opening anionic polymerization (ROAP) [49]. The controlled mesylation on hPG **1** yielded **2a–c** (95%, 57% and 30% of functionalization, respectively (for details see Supporting Information File 1)), which were converted to the corresponding azides **3a–c** [63–64]. Azide **6** was prepared according to well-established protocols [65]. Consequently, we adopted the Sharpless–Fokin modification for the Huisgen azide–alkyne cycloaddition [66] to achieve the final immobilization of the modified imidazolidin-4-one onto the hyperbranched polymer and on the G1 dendron [65]. The progress of the reaction was monitored by IR spectroscopy and TLC. Purification of the products **4a–c** was carried out by washing with aqueous saturated EDTA solution followed by dialysis in methanol/chloroform mixture for 24 h, and then in methanol and chloroform, respectively, for additional 12 h each. The catalyst structures were confirmed by ^1^H and ^13^C NMR spectroscopy and the functionalization degrees of **4a–c** were determined by correlating the aromatic with the polyglycerol backbone protons (for details see Supporting Information File 1).

**Scheme 2:**
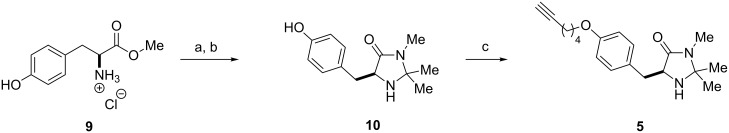
Synthesis of tyrosine-based imidazolidin-4-one **5**. Reaction conditions: (a) **9** (1.0 equiv), MeNH_2_ (5.0 equiv), EtOH, 25 °C, 20 h. (b) PTSA (0.01 equiv), acetone, MeOH, reflux, 18 h, 79% (2 steps). (c) **10** (1.0 equiv), NaH (1.1 equiv), 6-chloro-1-hexyne (1.3 equiv), TBAI (0.01 equiv), DMF, 25 °C, 16 h, 88%.

The synthesis of modified imidazolidin-4-one **5** started with (*S*)-tyrosine methyl ester hydrochloride (**9**). Following a protocol by Zhang and co-workers [58], **10** was obtained in good yield and subsequent anchoring of the linker was realized through *O*-alkylation on phenol **10**, leading to linkable catalyst **5** in excellent yield (Scheme 2).

The reactivity of the multivalent catalysts **4a–c** was investigated in the Friedel–Crafts alkylation of *N*-methylpyrrole (**11**) with α,β-unsaturated aldehydes reported by MacMillan [53]. To make the results comparable, we normalized the loading of the multivalent catalysts **4a–c** with respect to the number of single anchored imidazolidin-4-ones. Therefore, a constant number of catalytic units for each degree of functionalization was maintained. Initially, we decided to perform the reaction using *trans*-cinnamaldehyde (**12**) as a model substrate and trifluoroacetic acid (TFA) as an additive. In a preliminary survey on the water influence, a catalyst loading of 3.5 mol % in THF was selected to allow **4a** and **4b** to operate under homogeneous conditions, while in the same solvent **4c** proved to be less soluble (Table 1).

**Table 1:** Initial screening on the Friedel–Crafts alkylation of *N*-methylpyrrole (**11**) with *trans*-cinnamaldehyde (**12**).^a^

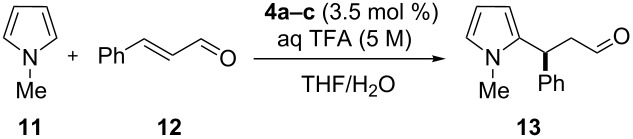				

Entry	Catalyst	THF/H_2_O (v/v)	Yield (%)^b^	ee (%)^c^

1	PG-95 (**4a**)	100:0	38	66
2	PG-57 (**4b**)	100:0	56	69
3	PG-30 (**4c**)	100:0	26	56
4	PG-95 (**4a**)	95:5	62	68
5	PG-57 (**4b**)	95:5	68	66
6	PG-30 (**4c**)	95:5	32	59
7	PG-95 (**4a**)	90:10	42	59
8	PG-57 (**4b**)	90:10	38	60
9	PG-30 (**4c**)	90:10	45	54
10	PG-95 (**4a**)	0:100	–^d^	–
11	PG-57 (**4b**)	0:100	–^d^	–
12	PG-30 (**4c**)	0:100	–^d^	–

^a^Reaction conditions: *trans*-cinnamaldehyde (**12**, 0.25 mmol, 1.0 equiv), *N*-methylpyrrole (**11**, 1.25 mmol, 5.0 equiv), catalyst **4a–c** (3.5 mol %), aq TFA (5 M; 3.5 mol %), 0.63 M with respect to *trans*-cinnamaldehyde (**12**), 25 °C, 20 h. ^b^Isolated yield. ^c^Determined by chiral GC. ^d^Complex mixture of products.

Moderate conversion of **13** were achieved using only THF as a solvent and in presence of substoichiometric amounts of water (0.4 equiv) [41]. Addition of water as co-solvent proved beneficial for the formation of product **13**. Notably, PG-95 (**4a**) and PG-57 (**4b**) exhibited comparable trends and the best yield and ee were observed when 5 vol % of water was added to the reaction mixture (Table 1, entries 4 and 5). Increasing the water content to 10 vol % resulted in incomplete conversion to **13** and lower ee values of the product (Table 1, entries 7 and 8). In case of the more hydrophilic PG-30 (**4c**) the activity increased with the amount of water in the reaction medium; yields and selectivities remained moderate. Attempts to carry out the reaction in water as the only solvent were unsuccessful (Table 1, entries 10–12). As expected, the outcomes of this reaction were strongly dependent on the solvent/water ratio and the catalysts **4a–c** exhibited different activity with changes on the degrees of functionalization. In general, catalysts **4a** and **4b** were found to be more efficient in comparison to the less functionalized **4c**. Probably, the poor ability of **4c** to catalyze the model transformation might be explained by its low solubility in the reaction medium, most likely due to the large number of free hydroxy groups on the periphery of the multivalent catalyst. Instead, catalysts **4a–c** demonstrated to be completely soluble in chloroform and methanol. Unfortunately, the use of these solvents led to decreased yields and selectivities of **13**. Therefore, we decided to further investigate the superior catalysts PG-95 (**4a**) and PG-57 (**4b**) in THF/H_2_O mixture.

As reported in the literature, immobilization of chiral imidazolidin-4-ones on polymeric support might affect the formation of the desired products and lead to decreased enantioselectivities [58]. Indeed, in all the experiments reported in Table 1 the enantiomeric excess of **13** was lower compared to MacMillan’s original experiments [53]. In an attempt to improve the enantiomeric ratios, we studied the influence of temperature using the optimized conditions obtained for **4a** and **4b** in Table 1 (for results, see Table 2).

**Table 2:** Influence of temperature in the Friedel–Crafts alkylation.^a^

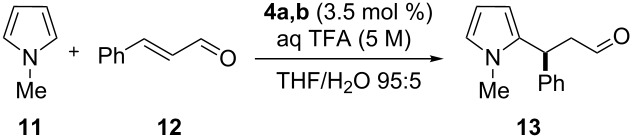					

Entry	Catalyst	*T* (°C)	*t* (h)	Yield (%)^b^	ee (%)^c^

1	PG-95 (**4a**)	25	20	62	68
2	PG-57 (**4b**)	25	20	68	66

3	PG-95 (**4a**)	4	35	60	68
4	PG-57 (**4b**)	4	35	64	68
5	PG-95 (**4a**)	–24	48	46	76
6	PG-57 (**4b**)	–24	48	25	78

^a^Reaction conditions: *trans*-cinnamaldehyde (**12**, 0.25 mmol, 1.0 equiv), *N*-methylpyrrole (**11**, 1.25 mmol, 5.0 equiv), catalyst **4a,b** (3.5 mol %), aq TFA (5 M; 3.5 mol %), THF/H_2_O 95:5 (v/v), 0.63 M with respect to *trans*-cinnamaldehyde (**12**). ^b^Isolated yield. ^c^Determined by chiral GC.

To our dismay, running the transformation at lower temperatures did not lead to any significant improvements, although slight changes were observed. Carrying out the reactions at 4 °C gave similar ee values (Table 2, entries 3 and 4), whereas at −24 °C the alkylation led to marginally increased selectivities, at the cost of a drop in the yield (Table 2, entries 5 and 6). Nevertheless, the observed enantiomeric excess of the product **13** is still low when compared with those (93% ee, at −30 °C) originally reported in the case of the traditional (*S*)-phenylalanine-based imidazolidin-4-one [53].

Using the optimized solvent system (Table 1), we then turned our attention to study the catalyst loading and further prove the efficiency of multivalent **4a** and **4b** (Table 3).

**Table 3:** Catalyst loading study.^a^

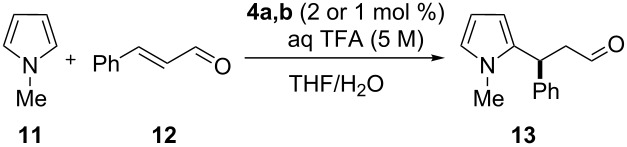					

Entry	Catalyst	Load. (mol %)	THF/H_2_O (v/v)	Yield (%)^b^	ee (%)^c^

1	PG-95 (**4a**)	2	95:5	43	59
2	PG-57 (**4b**)	2	95:5	62	64
3	PG-95 (**4a**)	2	97:3	66	68
4	PG-57 (**4b**)	2	97:3	65	67
5	PG-95 (**4a**)	1	98.5:1.5	46	67
6	PG-57 (**4b**)	1	98.5:1.5	50	74

^a^Reaction conditions: *trans*-cinnamaldehyde (**12**, 0.50 mmol, 1.0 equiv), *N*-methylpyrrole (**11**, 2.50 mmol, 5.0 equiv), catalyst **4a,b** (2 or 1 mol %), aq TFA (5 M; 2 mol %, entries 1–4 or 1 mol %, entries 5 and 6), 0.63 M with respect to *trans*-cinnamaldehyde (**12**), 25 °C, 48 h. ^b^Isolated yield. ^c^Determined by chiral GC.

Initial attempts with 2 mol % of the multivalent **4a** and **4b**, using 5 vol % of water in THF led to the isolation of **13** in moderate yield and slightly lower enantioselectivies, a result even more pronounced in the case of PG-95 (**4a**) (Table 3, entries 1 and 2). Next, we questioned if in addition to a catalyst loading reduction also a concomitant reduction of the water amount was necessary to maintain yield and enantiomeric ratio. Consistently, we reduced the water amount from 5 to 3 vol % and observed higher conversion to **13** and improved ee values (Table 3, entries 3 and 4). Therefore, in the following experiments the catalyst/water ratio was kept constant. The excellent efficiency of the catalyst was confirmed with moderate to good yields of **13** even though using 1 mol % of **4a** and **4b**, respectively (Table 3, entries 5 and 6). Considering, for the supported case, a typical catalyst loading for this transformation to be 10 mol % in order to achieve good conversion [62], the loadings reported in Table 3 could be decreased by one order of magnitude.

After solvent and temperature screening, our studies were focused on dilution experiments (Table 4).

**Table 4:** Dilution experiments.^a^

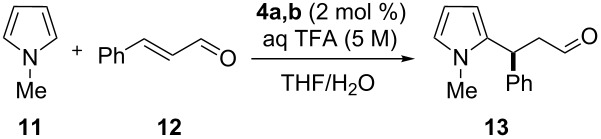					

Entry	Catalyst	Conc. (M)^b^	THF/H_2_O (v/v)	Yield (%)^c^	ee (%)^d^

1^e^	PG-95 (**4a**)	0.63	97:3	66	68
2^e^	PG-57 (**4b**)	0.63	97/3	65	67

3	PG-95 (**4a**)	0.30	98.5:1.5	70	68
4	PG-57 (**4b**)	0.30	98.5:1.5	87	70
5	PG-95 (**4a**)	0.10	99.5:0.5	<1^f^	n.d.^g^
6	PG-57 (**4b**)	0.10	99.5:0.5	29^f^	n.d.^g^

^a^Reaction conditions: *trans*-cinnamaldehyde (**12**, 0.25 mmol, 1.0 equiv), *N*-methylpyrrole (**11**, 1.25 mmol, 5.0 equiv), catalyst **4a,b** (2 mol %), aq TFA (5 M; 2 mol %), 25 °C, 48 h. ^b^With respect to *trans*-cinnamaldehyde (**12**). ^c^Isolated yield. ^d^Determined by chiral GC. ^e^*trans-*Cinnamaldehyde (**12**, 0.50 mmol, 1.0 equiv), *N*-methylpyrrole (**11**, 2.50 mmol, 5.0 equiv). ^f^Determined by ^1^H NMR. ^g^n.d. = not determined.

The best yield and enantioselectivity of **13** was obtained using PG-57 (**4b**) and lowering the concentration from 0.63 to 0.30 M (Table 4, entry 4). Contrarily, PG-95 (**4a**) did not lead to any appreciable improvement (Table 4, entry 3). By reducing the concentration to 0.10 M, **4b** gave only poor to moderate yields while the efficiency of **4a** decreased even more sharply and only traces of product **13** were observed (Table 4, entries 5 and 6). On the other hand, the enantioselectivities remained unchanged passing from concentration of 0.63 M to more diluted conditions (0.30 M). This outcome might be attributed to the constant local neighborhood in the polymer periphery where the catalytic centers are located, therefore the concentration may not affect the chiral induction [24].

After the completion of our systematic optimization of the reaction parameters using the model transformation, the most active catalyst **4b** was selected for a screening of different enals in the alkylation reaction of *N*-methylpyrrole (**11**). A study on the substrate scope was further carried out under the established conditions (see Table 4, entry 4). A variety of substituted α,β-unsaturated aldehydes **14a–e** was employed using 2 mol % PG-57 (**4b**) in THF/H_2_O 98.5:1.5 (v/v) (Table 5).

**Table 5:** Substrate scope.^a^

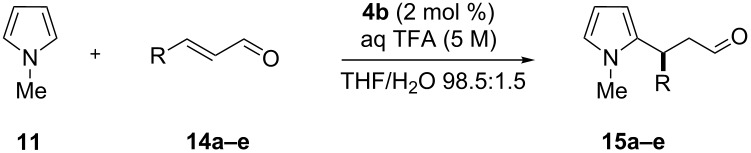							

Entry	Substrate		Product		*t* (h)	Yield (%)^b^	ee (%)^c^

1	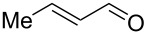	**14a**	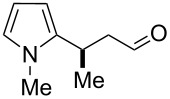	**15a**	24	86	69
2	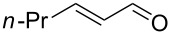	**14b**	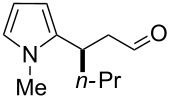	**15b**	24	83	68
3	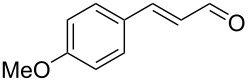	**14c**	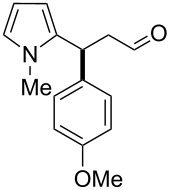	**15c**	48	80	56
4	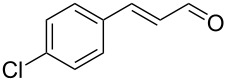	**14d**	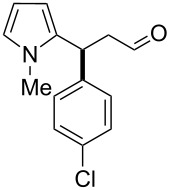	**15d**	48	86	71
5	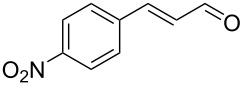	**14e**	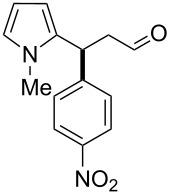	**15e**	48	99	78

^a^Reaction conditions: aldehyde **14a–e** (0.25 mmol, 1.0 equiv), *N*-methylpyrrole (**11**, 1.25 mmol, 5.0 equiv), catalyst **4b** (2 mol %), aq TFA (5 M; 2 mol %), THF/H_2_O 98.5:1.5 (v/v), 0.30 M with respect to aldehyde **14a–e**, 25 °C. ^b^Isolated yield. ^c^Determined by chiral GC.

Multivalent catalyst **4b** showed good to excellent activities in a range of substrates and moderate to good enantiomeric ratios for the formation of products **15a–e**, as shown in Table 5. Electron-deficient aromatic enals **14d,e** afforded higher yields and selectivities, confirming the strong influence of the substituent (Table 5, entries 4 and 5). Contrarily, aliphatic enals **14a,b** were well-tolerated and the outcomes were not affected significantly (Table 5, entries 1 and 2).

In our studies on the utilization of hPG as a soluble support in organocatalysis, hyperbranched PG-95 (**4a**) and PG-57 (**4b**) were finally compared with the monovalent G1-dendron imidazolidin-4-one **8** previously prepared and the original MacMillan’s first generation catalyst **16** using the optimum conditions (Table 6).

**Table 6:** Comparison of hPG catalysts **4a,b** with monovalent analogue **8** and MacMillan’s first generation **16**.^a^

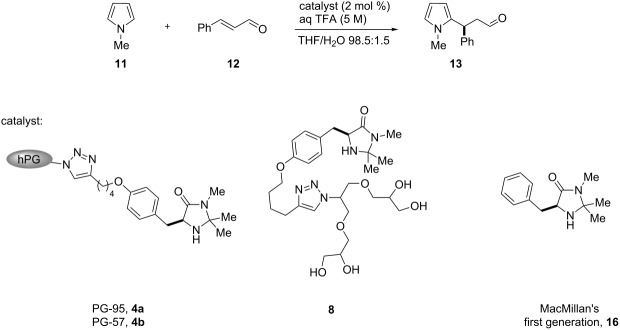			

Entry	Catalyst	Yield (%)^b^	ee (%)^c^

1	PG-95 (**4a**)	70	68
2	PG-57 (**4b**)	87	70
3	**8**	83	67
4	**16**	64	77

^a^Reaction conditions: *trans*-cinnamaldehyde (**12**, 0.25 mmol, 1.0 equiv), *N*-methylpyrrole (**11**, 1.25 mmol, 5.0 equiv), cat. (2 mol %), aq TFA (5 M; 2 mol %), THF/H_2_O 98.5:1.5 (v/v), 0.30 M with respect to *trans-*cinnamaldehyde (**12**), 25 °C, 48 h. ^b^Isolated yield. ^c^Determined by chiral GC.

As shown in Table 6, multivalent **4b** and monovalent **8** afforded similar results (Table 6, entries 2 and 3), probably due to their comparable high hydrophilicity. This outcome did not indicate additional cooperative effects between the active catalytic sites. Increased activities were observed compared to MacMillan’s catalyst **16**, albeit with lower enantioselectivity (Table 6, entries 2, 3 and 4). Catalyst **4a** showed turnover rates comparable with the traditional imidizolidin-4-one **16** (Table 6, entries 1 and 4). In conclusion, high- (PG-95, **4a**) or low- (PG-30, **4c**) loaded support were less active when compared to an intermediate degree of functionalization (PG-57, **4b**). In the case of PG-57 (**4b**) a good compromise between hydrophilicity and solubility was achieved. The results reported in Table 6 point out that catalyst **4b** was not suffering from diminished reactivity as often observed with immobilizations. Additionally the polymeric support was found to be responsible for enhanced reactivity with respect to the original imidazolidin-4-one **16**. The presence of anchimeric assistance by hydroxy groups, in the hydrolysis step of the iminium intermediate, might account for the observed improved turnover rates.

To complete our studies on the generality of hPG catalysts, finally, recycling of the polymer was studied. Heterogeneous catalysis allowed for simple separations of the immobilized species from the reaction media. Indeed, working under homogeneous conditions did not enable separation by simple filtration. On the other hand, the multivalent catalysts **4a** and **4b** showed poor solubility in solvents with low polarity, thus allowing for an easy recovery in 60–70% yield after selective precipitation using Et_2_O. The utility of our soluble support was examined in the catalytic efficiency of recovered PG-57 (**4b**) (Table 7).

**Table 7:** Catalyst recycle.^a^

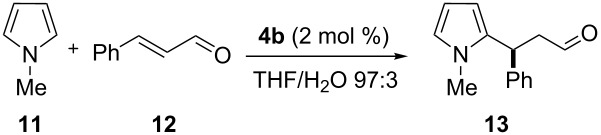			

Entry	Cycle	Yield (%)^b^	ee (%)^c^

1^d^	1	65	67
2	2	54	65
3^d^	3	45	65

^a^Reaction conditions: *trans-*cinnamaldehyde (**12**, 1.0 equiv), *N*-methylpyrrole (**11**, 5.0 equiv), catalyst **4b** (2 mol %), THF/H_2_O 97:3 (v/v), 0.63 M with respect to *trans*-cinnamaldehyde (**12**), 25 °C, 48 h. ^b^Isolated yield. ^c^Determined by chiral GLC. ^d^Aq TFA (5 M; 2 mol %) was added to the reaction mixture.

Catalyst **4b** was used three times in the asymmetric alkylation reaction. The experiment showed constant enantiomeric ratios although decreased activity and yields were observed. The lower yields exhibited after each cycle might be attributed to the decreased solubility of the recovered polymer. For the same reason, early attempts using the optimized parameters (conc. 0.30 M) were not successful; therefore the same PG-57 (**4b**) was subjected to more concentrated conditions (conc. 0.63 M). Moreover, addition of the acidic co-catalyst was crucial to establish the reactivity of the imidazolidin-4-one in the third cycle. Attempts to elucidate the reason of the decreased reactivity and analysis of the recovered polymer by ^1^H NMR indicated the leakage of the imidazolidin-4-one moiety. Nevertheless, studies focussing on improved catalyst stability and recycling are in progress.

## Conclusion

In summary, we have successfully employed a CuAAC strategy in the first immobilization of a chiral imidazolidin-4-one onto hyperbranched polyglycerol support and examined its efficiency in organocatalysis. Catalyst **4c** proved to be less soluble in the reaction media compared to **4a** and **4b**, and showed poor activity and selectivity. The soluble polymers **4a** and **4b** enabled homogeneous reactions without loss of efficiency due to immobilization. The activity of multivalent catalyst **4a** was comparable with that exhibited by the traditional MacMillan’s catalyst, while **4b** was shown to be superior. Nevertheless, erosion in enantioselectivity was observed, probably as a consequence of high local concentration effects on the periphery of the dendritic architecture, where the catalytic sites are located. The novel multivalent system **4b** achieved good conversion to afford product **13**, even with low polymer loading (1 mol %) compared to common loadings of 10 mol % required for the supported imidazolidin-4-ones. Moreover, **4b** was shown to be well-tolerated in a range of α,β-unsaturated aldehydes. The improved efficiency shown by **4b** might derive from an anchimeric assistance in the hydrolysis step of the iminium ion. Interestingly, the presence of such an effect might offer opportunities for further studies. One of the advantages of the multivalent catalyst **4b** was demonstrated to be its easy separability from the reaction media and its reuse for three consecutive times, whereas further investigations will be necessary on recycling of the polymeric support.

## Supporting Information

File 1 Experimental procedures, analytical data, copies of NMR spectra and GC reports.
