# Supplementary material for: Multivalent polyglycerol supported imidazolidin-4-one organocatalysts for enantioselective Friedel–Crafts alkylations
Source: Beilstein J Org Chem. 2015 May 12;11:730–8. doi: 10.3762/bjoc.11.83 (PMC4463494; doi:10.3762/bjoc.11.83)
Supplement: File 1 — Experimental procedures, analytical data, copies of NMR spectra and GC reports. [file Beilstein_J_Org_Chem-11-730-s001.pdf]

Supporting Information

for

Multivalent polyglycerol supported imidazolidin-4-one organocatalysts for enantioselective Friedel–Crafts slkylations

Tommaso Pecchioli, Manoj Kumar Muthyala, Rainer Haag* and Mathias Christmann*

Address: [1]Institut für Chemie und Biochemie, Freie Universität Berlin, Takustraße 3, 14195 Berlin, Germany

Email: Rainer Haag - haag@chemie.fu-berlin.de; Mathias Christmann - mathias.christmann@fu-berlin.de

*Corresponding author

Experimental procedures, analytical data, copies of NMR spectra and GC reports

Contents

1. **General methods**......S2
2. **Experimental procedures**......S3
3. **General procedure for the Friedel–Crafts alkylations**......S15
4. **Friedel–Crafts alkylation products**......S16
5. **Spectra of compounds**......S21
6. **GC reports**......S38
7. **References**......S44

1. General methods

Commercial reagents were used as received. All reactions were carried out under magnetic stirring and were monitored by TLC analysis on 0.20 mm silica gel plates (Macherey-Nagel G/UV_{254}). Column chromatography was carried out on silica gel 60 M (0.04–0.063 mm) Macherey-Nagel. Dialysis was performed in benzoylated cellulose tubes from Sigma-Aldrich (D7884-10FT, width: 32 mm, molecular weight cut-off (MWCO) 2000 g·mol^{-1}. Yields refer to spectroscopically and analytically pure compounds unless otherwise stated. ^{1}H NMR and ^{13}C NMR spectra were recorded on Bruker (ECP 400, AC 500, AV 700) or JEOL (ECX 400, Eclipse 500) instruments. Chemical shifts are reported relative to $CDCl_3$ (^{1}H: δ = 7.24 ppm; ^{13}C: δ = 77.23 ppm), DMSO-d_6 (^{1}H: δ = 2.50 ppm; ^{13}C: δ = 39.51 ppm) or acetone-d_6 (^{1}H: δ = 2.05 ppm; ^{13}C: δ = 29.92 ppm). Integrals are in accordance with assignments, coupling constants are given in Hz. For detailed peak assignments 2D spectra were recorded where necessary (COSY, DEPT, HSQC, HMQC, HMBC and NOESY). IR spectra were recorded ona Perkin–Elmer Spectrum BX FTIR System spectrophotometer JASCO FT/IR–4100. HRMS analyses were performed on a Varian Inc. Ionspec QFT-7 (ESI-TOF, 4 µL/min, 1.0 bar, 4 kV). Optical rotation measurements were performed on a P-2000 polarimeter from Jasco in a 1 dm optical-path length cell with the frequency of the Na_D line measured at the temperature and concentration (in g/100 mL) indicated. The enantiomeric excess was determined by chiral GC: Agilent 6850 Series II GC System equipped with Hydrodex-*β*-TBDAc column or Agilent 7890B equipped with Lipodex E column, the standards were prepared using racemic 5-benzyl-2,2,3-trimethylimidazolidin-4-one as catalyst.

2. Experimental procedures

General procedure for the synthesis of compound 10[1]

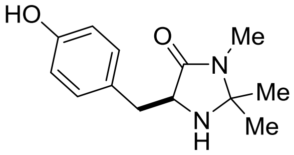

(*S*)-5-(4'-Hydroxylbenzyl)-2,2,3-trimethylimidazolidin-4-one (10). To an ethanolic solution of $MeNH_2$ (8.0 M in EtOH; 69 mL, 550 mmol, 5.0 equiv) (*S*)-tyrosine methyl ester hydrochloride (**9**, 25.5 g, 110 mmol, 1.0 equiv) was added and the solution was stirred for 20 h at 25 °C. After completion of the reaction the organic solvents were removed under reduced pressure, the residue was re-suspended in THF and again concentrated. To remove excess $MeNH_2$ the THF addition–removal cycle was repeated several times. The white solid thus obtained was used in the next step without further purification. The crude product and *p*-toluenesulfonic acid (209 mg, 1.10 mmol, 0.01 equiv) were dissolved in mixture of anhyd. MeOH (200 mL) and anhyd. acetone (40 mL) and the mixture was refluxed for 18 h. After completion, the reaction mixture was cooled to room temp., all solvents were removed under reduced pressure, and the so-obtained residue was purified by column chromatography on silica gel (CH_2Cl_2/MeOH 20:1) to yield **10** (20.4 g, 79%). ^{1}H-NMR ($CDCl_3$, 700 MHz): δ = 6.99 (d, *J* = 8.5 Hz, 2H; Ar-H), 6.72 (d, *J* = 8.5 Hz, 2H; Ar-H), 3.75 (t, *J* = 5.3 Hz, 1H; CH), 2.98 (d, *J* = 5.4 Hz, 2H; Bn-H), 2.72 (s, 3H; N-Me), 1.25 (s, 3H; Me), 1.14 ppm (s, 3H; Me); ^{13}C-NMR ($CDCl_3$, 175 MHz): δ = 173.9, 155.9, 130.7, 127.3, 115.9, 76.1, 59.4, 35.8, 27.1, 25.5, 25.1 ppm.

Synthesis of compound 5[1]

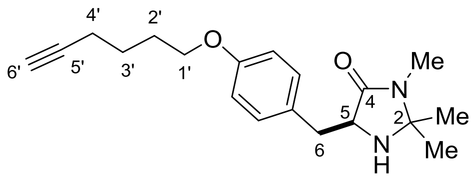

(*S*)-5-(*p*-(Hex-5'-yn-1'-yloxy)benzyl)-2,2,3-trimethylimidazolidin-4-one (5). NaH (60% in mineral oil; 282 mg, 7.02 mmol, 1.1 equiv) was added to solution of **10** (1.50 g, 6.40 mmol, 1 equiv) in anhyd. DMF (5 mL) at 0 °C. After 30 min of stirring at 0 °C, TBAI (23.0 mg, 0.06 mmol, 0.01 equiv) and 6-chloro-1-hexyne (1.01 mL, 8.32 mmol, 1.3 equiv) were added to the reaction and the mixture was allowed to warm to 25 °C and stirred for an additional 16 h. After this time, MeOH (1 mL) and H_2O (5 mL) were added and the mixture was extracted with CH_2Cl_2 (3 × 20 mL). The combined organic layers were dried over anhyd. Na_2SO_4, filtered, and concentrated under reduced pressure. The residue was purified by column chromatography on silica gel (pentane/EtOH/EtOAc 10:2:1) to yield **5** (1.78 g, 88%) as white solid. m.p.: 45–47 °C; R_f = 0.14 (pentane/EtOH/EtOAc 10:2:1); $[\alpha]_D^{24}$ = –69.7(*c* = 0.94 in $CHCl_3$); ^{1}H-NMR ($CDCl_3$, 700 MHz): δ = 7.10 (d, *J* = 8.7 Hz, 2H; Ar-H), 6.79 (d, *J* = 8.7 Hz, 2H; Ar-H), 3.93 (t, *J* = 6.3 Hz, 2H; H-1'), 3.72 (dd, *J* = 5.9, 5.0 Hz, 1H; H-5), 3.04–2.96 (AB system, *J* = 14.4, 6.3 Hz, 2H; Bn-H), 2.72 (s, 3H; N-Me), 2.24 (td, *J* = 7.1, 2.7 Hz, 2H; H-4'), 1.95 (t, *J* = 2.7 Hz, 1H; H-6'), 1.89–1.85 (m, 2H; H-2'), 1.71–1.66 (m, 2H; H-3'), 1.24 (s, 3H; Me), 1.14 ppm (s, 3H; Me); ^{13}C-NMR ($CDCl_3$, 175 MHz): δ = 173.4 (C-4), 157.9 (Ar-C), 130.5 (2Ar-CH), 128.8 (Ar-C), 114.6 (2Ar-CH), 84.1 ($\underline{C}$≡CH), 75.5 (C-2), 68.7 (C≡$\underline{C}$H), 67.2 (C-1'), 59.3 (C-5), 36.1 (Bn-C), 28.3 (C-2'), 27.2 (N-Me), 25.2 (2xMe), 25.0 (C-3'), 18.1 ppm (C-4'); IR ($CDCl_3$): $\tilde{\nu}$ = 3304, 3286, 2974, 2943, 2871, 1688, 1612, 1580, 1511, 1474, 1428, 1398, 1368, 1244, 1178, 1148,

1054, 1032, 822 cm^{-1}; HRMS (ESI) : m/z : calcd for $C_{19}H_{26}N_2O_2+H^+$: 315.2067; found: 315.2075 $[M+H^+]$.

Synthesis of hPG-OH 1[2]

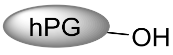

Hyperbranched polyglycerol (hPG) **1** with M_n= 9.000 g/mol^{-1} (loading OH = 13.5 mmol/g, PDI = 1.87) was synthesized by a one-step ring opening anionic polymerization (ROAP) method, according to the earlier reported methods.[2] 1,1,1-Tris(hydroxymethyl)propane (TMP) was used as the starter in ROAP. Azide functionalized hPG were synthesized according to previously reported procedures.[3-4]

Synthesis of hPG-OMs 2a–c[3,4]

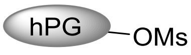

General procedure: Mesylation of hyperbranched polyglycerol **1** was carried out under an inert gas atmosphere and exclusion of moisture. In a two-necked 1 L flask, hyperbranched polyglycerol **1** (13.51 mmol OH-groups) was dissolved in anhyd. pyridine (60 mL). The resulting solution was stirred at 25 °C for 10 min and then cooled to 0 °C in an ice bath. A solution of methanesulfonyl chloride (1.2 equiv, with respect to functionalization degree) in anhyd. pyridine (20 mL) was added dropwise to the reaction mixture and stirring at 25 °C was continued for 16 h. The reaction mixture was then filtered and the solvent was removed under reduced pressure.

The degrees of functionalization were confirmed by ^{1}H NMR of the crude products correlating the CH_3-Ms with polyglycerol backbone protons.

hPG-OMs (>95%), 2a

Reaction conditions were as described above, using **1** (1.0 g). The crude product was washed with cold H_2O (3 × 10 mL), further dissolved and dialyzed in acetone for 72 h to give pure **2a** (1.52 g, 76% yield). ^{1}H-NMR (DMSO-d_6, 400 MHz): δ = 5.09–4.86 (functionalized secondary PG-groups), 4.58–4.35 (functionalized primary PG-groups), 4.05–3.45 (PG backbone), 3.27–3.11 (br s; Ms), 1.49–1.39 (m; C$\underline{CH_2}$$CH_3$ of starter), 0.91 ppm (t; $CCH_2$$\underline{CH_3}$, of starter).

hPG-OMs (57%), 2b

Reaction conditions were as described above, using **1** (1.0 g). The crude product was dissolved in an acetone/H_2O mixture (1:1 v/v) and dialyzed in the same mixture for 72 h to give pure **2b** (1.31, 82% yield). ^{1}H-NMR (acetone-d_6, 400 MHz): δ = 5.08–4.85 (functionalized secondary PG-groups), 4.42–4.17 (functionalized primary PG-groups), 4.05–3.75 (PG backbone), 3.25–3.12 (br s; Ms), 1.42–1.34 (m; C$\underline{CH_2}$$CH_3$ of starter), 0.87 ppm (t; $CCH_2$$\underline{CH_3}$, of starter).

hPG-OMs (30%), 2c

Reaction conditions were as described above, using **1** (1.0 g). The crude product was dissolved in a MeOH/H_2O mixture (1:1 v/v) and dialyzed in the same mixture for 72 h to give pure **2c** (1.12, 87% yield). ^{1}H-NMR (DMSO-d_6, 400 MHz): δ = 5.11–4.81 (functionalized secondary PG-groups), 4.30–4.21 (functionalized primary PG-groups), 4.15–3.45 (PG backbone), 3.27 (br s; Ms), 1.43–1.31 (m, C$\underline{CH_2}$$CH_3$ of starter), 0.90 ppm (t, $CCH_2$$\underline{CH_3}$, of starter).

Synthesis of hPG-N_3 3a–c[3,4]

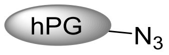

General procedure: To a homogeneous mixture of *O*-mesylpolyglycerol **2a–c** (1 equiv) in DMF (15 mL), NaN_3 (3 equiv) was added and the resulting suspension was heated at 65 °C for 72 h. After completion of the reaction, the mixture was cooled to room temp. and filtered through Celite® to remove excess NaN_3. The filtrate was concentrated under reduced pressure at a temperature below 40 °C and handled with a plastic spatula to avoid a potentially explosive degradation of the polyazide.

hPG-N_3 (95%), 3a

Reaction conditions were as described above, using **2a** (1.52 g). The crude product was dissolved in $CHCl_3$ and extracted four times with H_2O. The organic phase was dried over anhyd. $MgSO_4$. To remove traces of DMF from the crude product an additional dialysis in a MeOH/$CHCl_3$ mixture (1:1 v/v) was performed to give pure **3a** (0.72 g, 72% yield). ^{1}H-NMR ($CDCl_3$, 400 MHz): δ = 4.04–3.35 (m; PG backbone), 1.45–1.33 (m; $C\underline{CH_2}CH_3$ of starter), 0.85 ppm (t; $CCH_2\underline{CH_3}$, of starter); ^{13}C-NMR ($CDCl_3$, 400 MHz): δ = 76.4–51.5 (PG backbone), 25.7 ($C\underline{CH_2}CH_3$ of starter), 8.8 ppm ($CCH_2\underline{CH_3}$ of starter); IR: $\tilde{\nu}$ = 2871, 2091 (N_3), 1447, 1345, 1267, 1174, 1099, 927 cm^{-1}.

hPG-N_3 (57%), 3b

Reaction conditions were as described above, using **2b** (1.31 g). The crude product was dissolved in acetone/H_2O mixture (1:1 v/v) and dialyzed in the same mixture for 72 h to give pure **3b** (0.78 g, 81% yield). ^{1}H-NMR ($CDCl_3$, 400 MHz): δ = 4.02–3.34 (m; PG backbone), 1.38–1.27 (m; $C\underline{CH_2}CH_3$ of starter), 0.79 (t; $CCH_2\underline{CH_3}$, of starter);

^{13}C-NMR ($CDCl_3$, 100 MHz): δ = 77.7–53.9 (PG backbone), 25.7 ($C\underline{CH_2}CH_3$ of starter), 8.0 ($CCH_2\underline{CH_3}$ of starter) ppm; IR: $\tilde{\nu}$ = 3398, 2871, 2096 (N_3), 1769, 1636, 1453, 1455, 1270, 1077, 991, 929 cm^{-1}.

hPG-N_3 (30%), 3c

Reaction conditions were as described above, using **2c** (1.12 g). The residue was purified by dialysis in H_2O for 48 h to give pure **3c** (0.78 g, 86% yield). ^{1}H-NMR (DMSO-d_6, 400 MHz): δ = 4.05–3.35 (m; PG backbone), 1.35–1.29 (m; $C\underline{CH_2}CH_3$ of starter), 0.77 ppm (t; $CCH_2\underline{CH_3}$ of starter); ^{13}C-NMR (DMSO-d_6, 100 MHz): δ = 78.5–54.0 (PG backbone), 24.1 ($C\underline{CH_2}CH_3$ of starter), 10.4 ppm ($CCH_2\underline{CH_3}$ of starter); IR: $\tilde{\nu}$ = 3382, 2871, 2359, 2341, 2098 (N_3), 2035, 1771, 1635, 1558, 1455, 1272, 1078, 932, 869, 671 cm^{-1}.

Synthesis of hPG-cat. 4a–c[5]

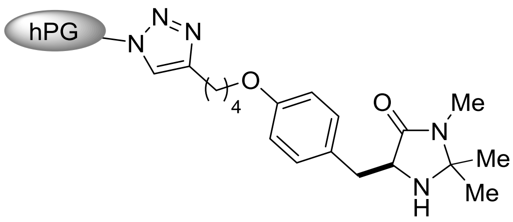

General procedure: hPG-azide **3a–c** (1.0 equiv) and alkyne **5** (2.0 equiv) were dissolved in a THF/H_2O mixture (3:1 v/v; 4 mL) and the resulting solution was degassed for 10 min. Sodium ascorbate (2.0 equiv) in H_2O (100 mg/mL) and $CuSO_4{\cdot}5H_2O$ (0.2 equiv) in H_2O (100 mg/mL) were mixed and the resulting solution was added dropwise to the solution of hPG-azide **3a–c** and alkyne **5**. The reaction mixture was stirred at 25 °C for app. 48 h and the progress of the reaction was monitored by infrared (IR) spectroscopy. After completion of the

reaction, the mixture was diluted with $CHCl_3$ and the resulting solution was washed with sat. aq. EDTA solution (2 × 10 mL), followed by water (2 × 10 mL), and dried over anhyd. Na_2SO_4. The compounds were further purified by dialysis MeOH/$CHCl_3$ mixture (1:1 v/v) 24 h, and then MeOH and $CHCl_3$, respectively, for 12 h each.

The degrees of functionalization were confirmed by ^{1}H NMR correlating the aromatic protons with polyglycerol backbone protons.

hPG-Cat (95%), 4a

Reaction conditions and purification methods were as described above, using **3a** (100 mg) to give pure **4a** (296 mg, 71% yield). ^{1}H-NMR ($CDCl_3$, 700 MHz): δ = 7.64–7.36 (m, 1H; triazole), 7.13–7.04 (m, 2H; Ar-H), 6.80–6.70 (m, 2H; Ar-H), 5.37–4.70 (functionalized secondary PG-groups), 4.67–4.38 (functionalized primary PG-groups), 4.31–3.15 (PG backbone), 3.92–3.84 (m, 2H; CH_2O-Ar), 3.74–3.65 (br s, 1H; HN<u>CH</u>CON[CH_3]), 3.07–2.98 (m, 1H; Bn-H), 2.97–2.88 (m, 1H; Bn-H), 2.76–2.63 (m, 5H; N-CH_3, triazole-CH_2-), 1.84–1.62 (m, 4H; [CH_2]$_2$-), 1.25 (s, 3H; Me), 1.17 ppm (s, 3H; Me); ^{13}C-NMR ($CDCl_3$, 175 MHz): δ = 173.5 (C=O), 157.8 (C-Ar), 147.7 (C_5-triazole), 130.5 (C-Ar), 129.1 (C-Ar), 122.0 (C-Ar), 114.5 (C-Ar), 75.6 (N<u>C</u>[CH_3])$_2$N), 72.9–68.6 (PG backbone), 67.4 (CH_2O-Ar), 59.4, 36.2, 29.0–28.7, 27.3, 26.1–25.9, 25.2 (2xMe), 24.3 ppm; IR: $\tilde{\nu}$ = 2929, 1682 (CONHCH_3), 1611, 1510, 1429, 1399, 1243, 1177, 1112, 811, 752 cm^{-1}.

hPG-Cat (57%), 4b

Reaction conditions and purification methods were as described above, using **3b** (100 mg) to give pure **4b** (121 mg, 40% yield). ^{1}H-NMR ($CDCl_3$, 700 MHz): δ = 7.57–7.40 (m, 1H; triazole), 7.09–7.07 (m, 2H; Ar-H), 6.75 (m, 2H; Ar-H), 5.18–4.68 (functionalized secondary PG-groups), 4.49–4.24 (functionalized primary PG-groups), 4.20–3.25 (PG backbone), 3.90–3.82 (m, 2H; CH_2OAr), 3.74–3.65 (br s, 1H; NH<u>CH</u>CON[CH_3]), 3.02–3.00 (m, 1H; Bn-H), 2.94–2.88 (m, 1H; Bn-H), 2.71–2.62 (m, 5H; N-CH_3, triazol-CH_2-), 1.76–1.67 (m, 4H; [CH_2]$_2$), 1.22 (s, 3H; Me), 1.14 ppm (s, 3H; Me); ^{13}C NMR ($CDCl_3$, 175 MHz): δ = 173.5 (C=O), 157.9 (C-Ar), 147.6 (C_5-triazole), 130.6 (C-Ar), 129.1–128.9 (C-Ar), 122.8–122.6 (C_4-triazole), 114.6 (C-Ar), 75.6 (N<u>C</u>[CH_3])$_2$N), 72.9–68.6 (PG backbone), 67.5 (CH_2OAr), 59.3, 36.2, 28.8, 27.2, 25.9, 25.2 (2xMe), 24.2 ppm; IR: $\tilde{\nu}$ = 3312, 2924, 1679 (CONHCH_3), 1611, 1510, 1428, 1398, 1388, 1296, 1241, 1176, 1110, 807, 664 cm^{-1}.

hPG-Cat (30%), 4c

Reaction conditions and purification methods were as described above, using **3c** (100 mg) to give pure **4c** (64.0 mg, 35% yield). ^{1}H-NMR ($CDCl_3$, 700 MHz): δ = 7.61–7.41 (m, 1H; triazole), 7.14–7.08 (m, 2H; Ar-H), 6.81–6.72 (m, 2H; Ar-H), 5.00–4.60 (functionalized secondary PG-groups), 4.44–4.32 (functionalized primary PG-groups), 4.28–3.15 (PG backbone), 3.87–3.80 (m, 2H; CH_2O-Ar), 3.03–3.01 (m, 1H; Bn-H), 3.76–3.67 (br s, 1H; NH<u>CH</u>CON[CH_3]), 2.94–2.89 (m, 1H; Bn-H), 2.76–2.63 (m, 5H; N-Me, triazol-CH_2-), 1.84–1.70 (m, 4H; [CH_2]$_2$), 1.27 (s, 3H; Me), 1.15 ppm (s, 3H; Me); ^{13}C-NMR ($CDCl_3$, 175 MHz): δ = 173.6 (C=O), 157.9 (C-Ar), 147.6 (C_5-triazole), 130.6 (C-Ar), 129.0–128.9 (C-Ar), 122.9–122.6 (C_4-Triazole), 114.6 (C-Ar),

75.6 (N<u>C</u>[CH_3])$_2$N), 72.9–68.6 (PG backbone), 67.5 (CH_2OAr), 59.3, 36.2, 28.8, 27.2, 25.9, 25.2 (2xMe), 24.2 ppm; IR: $\tilde{\nu}$ = 3357, 2869, 1675 (CONHCH$_3$), 1611, 1431, 1400, 1242, 1177, 1077, 808, 664 cm^{-1}.

Synthesis of compound 6

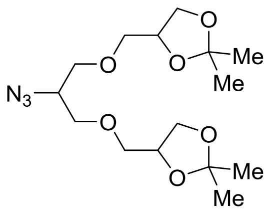

[G1]-N_3 was synthesized according to the earlier reported methods.[6]

Synthesis of compound 7

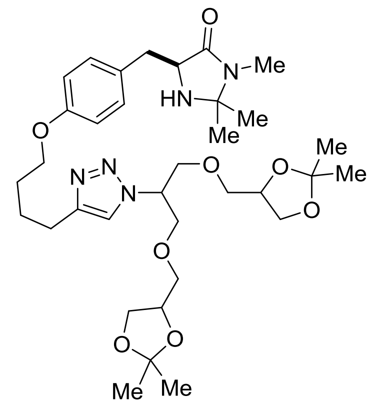

To a homogeneous solution of alkyne **5** (100 mg, 0.32 mmol, 1.0 equiv) and [G_1]-N_3 **6** (120 mg, 0.35 mmol, 1.1 equiv) in a THF/H_2O mixture (3:1 v/v; 4 mL), DIPEA (4.00 mg, 0.032 mmol, 0.1 equiv) was added. Stock solutions (100 mg/mL in H_2O) of sodium ascorbate (13.0 mg, 0.064 mmol, 0.2 equiv) and $CuSO_4 \cdot 5H_2O$ (8.00 mg, 0.032 mmol, 0.1 equiv) were added simultaneously to the reaction mixture which was further stirred at 25 °C for 12 h. After completion of the reaction, the resulting residue was diluted with H_2O and

extracted with CH_2Cl_2 (3 × 10 mL). The combined organic layers were washed consecutively with sat. aq EDTA solution (2 × 10 mL) and H_2O (3 × 10 mL), dried over anhyd. Na_2SO_4, and concentrated under reduced pressure. The resulting crude product was purified by column chromatography on silica gel (CH_2Cl_2/MeOH 20:1) to give pure **7** as a mixture of diastereoisomers (150 mg, 70% yield).

^{1}H-NMR (CD_3OD, 500 MHz): δ = 7.88–7.79 (m, 1H; triazole), 7.17–7.12 (m, 2H; Ar-H), 6.86–6.82 (m, 2H; Ar-H), 4.96–4.93 (m, 1H), 4.21–4.13 (m, 2H), 4.12–4.02 (m, 1H), 3.98–3.88 (m, 7H), 3.74 (dd, *J* = 7.8, 4.1 Hz, 1H), 3.64–3.58 (m, 2H), 3.54–3.43 (m, 4H), 3.03 (dd, *J* = 14.3, 4.2 Hz, 1H; Bn-H), 2.85 (dd, *J* = 14.3, 7.3 Hz, 1H; Bn-H), 2.78 (d, *J* = 7.0 Hz, 2H; triazole-CH_2), 2.75 (s, 3H; N-CH_3), 1.88–1.75 (m, 4H; -CH_2-), 1.36 (s, 3H; $OC(CH_3)_2$), 1.31–1.29 (m, 6H; $OC(CH_3)_2$), 1.23 (s, 3H; Me), 1.13 ppm (s, 3H; Me); ^{13}C-NMR (CD_3OD, 125 MHz): δ = 174.2, 158.2, 147.2, 130.2, 129.2, 121.9, 114.3, 109.2 (2C), 76.1, 74.7 (2C), 71.9, 71.6, 70.1, 70.0, 69.9, 67.3, 66.0, 59.6, 35.8, 28.5, 25.8, 25.7, 25.6, 24.7, 24.3 (2C), 24.2, 23.5 ppm; IR: $\tilde{\nu}$ = 2982, 2932, 2870, 1685 ($CONHCH_3$), 1611, 1581, 1550, 1510, 1473, 1455, 1427, 1397, 1380, 1369, 1241, 1177, 1145, 1111, 1078, 953, 838, 731 cm^{-1}; HRMS (ESI) calcd for $C_{34}H_{45}N_5O_8+H^+$: 660.3967; found 660.3970 $[M+H]^+$, 682.3748 $[M+Na]^+$.

Synthesis of compound 8[6]

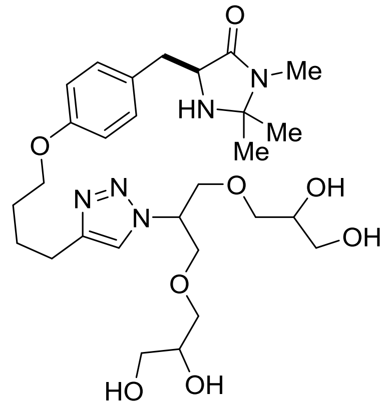

Ion exchange resin Dowex 50W (500 mg) was added to **7** (100 mg, 0.15 mmol) dissolved in MeOH (4 mL), and the mixture heated to reflux for 12 h. After cooling, Dowex 50W was filtered off and washed with a 6% solution of NH_3 in MeOH. The filtrate was concentrated under reduced pressure to yield **8** as a mixture of diastereoisomers (80.0 mg, 95% yield).

^{1}H-NMR (CD_3OD, 700 MHz): δ = 7.93–7.81 (m, 1H; triazole), 7.19–7.14 (m, 2H; Ar-H), 6.87 (dd, J = 8.8, 2.6 Hz, 2H; Ar-H), 5.00–4.94 (m, 1H), 4.66–4.48 (m, 1H), 4.04–3.88 (m, 4H), 3.82–3.42 (m, 12H), 3.05 (dd, J = 14.4, 4.2 Hz, 1H; Bn-H), 2.88 (dd, J = 14.4, 7.2 Hz, 1H; Bn-H), 2.77 (m, 5H), 1.95–1.77 (m, 4H), 1.29 (s, 3H; Me), 1.23 ppm (s, 3H; Me); ^{13}C-NMR (CD_3OD, 125 MHz): δ = 174.1, 158.1, 158.1, 147.4, 147.2, 130.2, 130.2, 129.1, 123.4, 123.3, 121.9, 121.9, 121.9, 115.0, 114.3, 77.7 (2C), 77.6 (2C), 76.1, 72.6 (2C), 72.4, 72.3, 71.3 (2C), 71.0 (2C), 70.8 (2C), 70.7, 70.0 (2C), 69.9 (2C), 67.2 (2C), 62.9 (3C), 62.8 (2C), 60.9 (3C), 60.1, 59.5, 51.4, 50.7, 50.6, 35.8, 35.6, 31.4, 28.7, 28.5, 28.4 (2C), 25.7, 25.6, 24.7, 24.6 (2C), 24.2, 23.5, 22.3, 13.0 ppm; IR: $\tilde{\nu}$ = 3339 (OH), 2928, 2870, 1736, 1657 (CO-NH-CH_3), 1611, 1580, 1549, 1510, 1444, 1404, 1298, 1241, 1177, 1111, 1024, 953, 820, 659 cm^{-1}; HRMS (ESI) calcd for $C_{28}H_{45}N_5O_8+H^+$: 580.3341; found: 580.3404 $[M+H]^+$, 602.318 $[M+Na]^+$.

General procedure for the synthesis of 16[7]

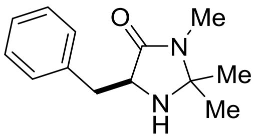

5-Benzyl-2,2,3-trimethylimidazolidin-4-one (16). Reaction conditions and work-up were as described above (Section 2 Supporting Information File 1, page S3), using (*S*)-phenylalanine methyl ester hydrochloride (1.08 g, 5.03 mmol, 1.0 equiv). The so-obtained crude product was purified by column chromatography on silica gel (EtOAc) to give pure **16** (790 mg, 72%).

^{1}H-NMR ($CDCl_3$, 500 MHz): δ = 7.24–7.14 (m, 5H), 3.72 (dd, *J* = 6.8, 4.5 Hz, 1H), 3.07 (dd, *J* = 14.2, 4.5 Hz, 1H), 2.94 (dd, *J* = 14.2, 6.8 Hz, 1H), 2.68 (s, 1H), 1.19 (s, 3H), 1.09 ppm (s, 3H); ^{13}C-NMR ($CDCl_3$, 125 MHz): δ= 173.4, 137.2, 129.5, 128.6, 126.8, 75.5, 59.3, 37.4, 27.3, 25.4, 25.2 ppm.

Synthesis of *trans*-*p*-methoxy-cinnamaldehyde (14c)[8]

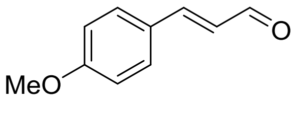

***trans*-*p*-Methoxy-cinnamaldehyde (14c)**. KO*t*-Bu (824 mg, 7.34 mmol, 2.0 equiv) was added to a suspension of (1,3-dioxan-2-ylmethyl)triphenylphosphonium bromide (3.47 g, 8.08 mmol, 2.2 equiv) in anhyd. THF (30 mL) at 0 °C and the mixture was stirred for 30 min at this temp. A solution of *p*-anisaldehyde (500 mg, 3.67 mmol, 1.0 equiv) in anhyd. THF (7 mL) was then slowly added, and the mixture was stirred for

1 h at 25 °C and then heated at reflux for an additional 24 h. Then, the reaction mixture was quenched by the addition of aq oxalic acid (8 g in 100 mL of H_2O) and stirred at 25 °C for an additional 16 h. Afterwards, the mixture was extracted with Et_2O (2 × 50 mL), the combined organic layers were washed consecutively with sat. aq $NaHCO_3$ (80 mL) and H_2O (80 mL), dried over anhyd. Na_2SO_4 and concentrated under reduced pressure. Purification of the residue by column chromatography on silica gel (pentane/EtOAc 7:1) afforded pure *trans*-*p*-methoxy-cinnamaldehyde (**14c**) as a yellow solid (595 mg, 83%).

^{1}H-NMR ($CDCl_3$, 400 MHz): δ = 9.62 (d, *J* = 7.8 Hz, 1H), 7.50 (d, *J* = 8.6 Hz, 2H), 7.40 (d, *J* = 15.8 Hz, 1H), 6.92 (d, *J* = 8.8 Hz, 2H), 6.58 (dd, *J* = 15.8, 7.8 Hz, 1H), 3.83 ppm (s, 3H); ^{13}C-NMR ($CDCl_3$, 175 MHz): δ = 193.7, 162.2, 152.7, 130.4, 126.8, 126.5, 114.6, 55.5 ppm.

3. General procedure for the Friedel–Crafts alkylations

A solution of the catalyst **4a–c** or **8** or **16** (**x** mol %) in the **solvent** indicated was treated with aq TFA (5 M; **x** mol %). The mixture was stirred at room temp. for 10 minutes, then the aldehyde **12** or **14a–e** (0.25 mmol, 1.0 equiv) was added at the desired temperature ***T***. After 5 minutes of stirring, *N*-methylpyrrole (**11**, 111 μL, 1.25 mmol, 5.0 equiv) was added and the reaction was stirred at the same temperature ***T***. Et_2O (3 mL) was added to the mixture and the catalyst was removed by filtration, washed several time with Et_2O, then recovered with CH_2Cl_2 and dried in vacuo for future use. The organic phase was concentrated under reduced pressure and the so-obtained residue was purified by silica gel chromatography (pentane/Et_2O) to afford the corresponding products.

4. Friedel–Crafts alkylations products

Synthesis of compound 13

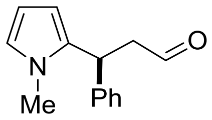

(*S*)-3-(1-Methyl-1*H*-pyrrol-2-yl)-3-phenylpropanal (13).[9] Reaction conditions and work-up were as described above, using commercially available *trans*-cinnamaldehyde (**12**, 31.5 μL) and *N*-methylpyrrole (**11**). Purification by column chromatography on silica gel (pentane/Et_2O 7:1) gave **13** (46.2 mg, 87%). ^{1}H-NMR ($CDCl_3$, 400 MHz): δ = 9.74 (t, *J* = 0.8 Hz, 1H), 7.30–7.13 (m, 5H), 6.55 (t, *J* = 2.0 Hz, 1H), 6.12–6.09 (m, 2H), 4.56 (t, *J* = 7.5 Hz, 1H), 3.31 (s, 3H), 3.15 (ddd, *J* = 17.2, 8.4, 2.0 Hz, 1H), 2.95 ppm (ddd, *J* = 17.2, 6.8, 1.6 Hz, 1H); ^{13}C-NMR ($CDCl_3$, 175 MHz): δ = 200.9, 142.5, 133.1, 128.8, 127.7, 126.8, 122.5, 106.7, 106.6, 50.1, 37.7, 33.9 ppm. The enantiomeric excess was determined by chiral GC on a Hydrodex-*β*-TBDAc column (120 ºC isotherm, 1.1 mL/min He): *S* isomer t_r = 185.45 min and *R* isomer t_r = 191.51 min. The absolute configuration was determined by reduction to the corresponding alcohol and comparison of the optical specific rotation with reported data.[9]

Synthesis of compound 15a

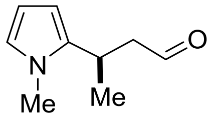

(*R*)-3-(1-Methyl-1*H*-pyrrol-2-yl)butanal (15a).[9] Reaction conditions and work-up were as described above, using commercially available predominantly *trans*-

crotonaldehyde (**14a**, 20.4 μL) and *N*-methylpyrrole (**11**). Purification by column chromatography on silica gel (pentane/Et_2O 9:1) gave **15a** (32.5 mg, 86%). ^{1}H-NMR ($CDCl_3$, 400 MHz): δ = 9.74 (t, *J* = 1.7 Hz, 1H), 6.53 (t, *J* = 2.2 Hz, 1H), 6.05 (t, *J* = 3.2 Hz, 1H), 5.88 (dd, *J* = 3.6, 2.0 Hz, 1H), 3.59 (s, 3H), 3.38 (q, *J* = 7.2 Hz, 1H), 2.79 (ddd, *J* = 17.2, 6.2, 1.5 Hz, 1H), 2.64 (ddd, *J* = 17.2, 7.9, 1.8 Hz, 1H), 1.27 ppm (d, *J* = 6.9 Hz, 3H); ^{13}C-NMR ($CDCl_3$, 175 MHz): δ = 201.7, 136.6, 121.6, 106.8, 104.2, 50.6, 33.6, 25.4, 21.4 ppm. The enantiomeric excess was determined by chiral GC on a Hydrodex-*β*-TBDAc column (130 °C isotherm, 1.1 mL/min He): *S* isomer t_r = 11.00 min and *R* isomer t_r = 11.48 min. The absolute configuration was determined by reduction to the corresponding alcohol and comparison of the optical specific rotation with reported data.[9]

Synthesis of compound 15b

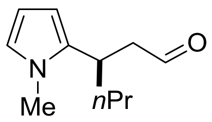

(*R*)-3-(1-Methyl-1*H*-pyrrol-2-yl)hexanal (15b).[10] Reaction conditions and work-up were as described above, using commercially available *trans*-2-hexenal (**14b**, 29.1 μL) and *N*-methylpyrrole (**11**). Purification by column chromatography on silica gel (pentane/Et_2O 8:1) gave **15b** (37.2 mg, 83%). ^{1}H-NMR ($CDCl_3$, 400 MHz): δ = 9.69 (t, *J* = 1.6 Hz, 1H), 6.50 (dd, *J* = 2.4, 1.6 Hz, 1H), 6.06 (t, *J* = 3.2 Hz, 1H), 5.87 (dd, *J* = 3.2, 1.6 Hz, 1H), 3.58 (s, 3H), 3.27 (q, *J* = 6.8 Hz, 1H), 2.73 (ddd, *J* = 17.2, 7.6, 1.6 Hz, 1H), 2.69 (ddd, *J* = 17.2, 6.8, 2.0 Hz, 1H), 1.61–1.55 (m, 2H), 1.30–1.22 (m, 2H), 0.87 ppm (t, *J* = 7.3 Hz, 3H); ^{13}C-NMR ($CDCl_3$, 175 MHz): δ = 202.1, 136.6, 121.2, 106.8, 104.8, 49.6, 38.6, 33.8, 30.5, 20.2, 14.0 ppm. The enantiomeric excess was

determined by chiral GC on a Hydrodex-β-TBDAc column (90 °C isotherm, 1.1 mL/min He): *R* isomer t_r = 111.97 min and *S* isomer t_r = 114.95 min. The absolute configuration was determined by reduction to the corresponding alcohol and comparison of the optical specific rotation with reported data.[10]

Synthesis of compound 15c

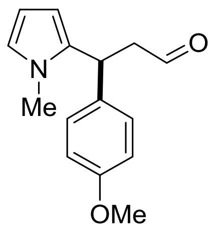

(*S*)-3-(4-Methoxyphenyl)-3-(1-methyl-1*H*-pyrrol-2-yl)propanal (15c).[10] Reaction conditions and work-up were as described above, using *trans*-*p*-methoxy-cinnamaldehyde (**14c**, 40.5 mg) and *N*-methylpyrrole (**11**). Purification by column chromatography on silica gel (pentane/Et_2O 6:1) gave **15c** (48.6 mg, 80%). ^{1}H-NMR ($CDCl_3$, 700 MHz): δ = 9.76 (t, *J* = 1.8 Hz, 1H), 7.07 (d, *J* = 8.7 Hz, 2H), 6.83 (d, *J* = 8.7 Hz, 2H), 6.57 (t, *J* = 2.1 Hz, 1H), 6.12 (dd, *J* = 3.5, 2.8 Hz, 1H), 6.09–6.08 (m, 1H), 4.53 (t, *J* = 7.5 Hz, 1H), 3.79 (s, 3H), 3.33 (s, 3H), 3.14 (ddd, *J* = 17.1, 7.9, 2.1 Hz, 1H), 2.95 ppm (ddd, *J* = 17.1, 7.9, 2.1 Hz, 1H); ^{13}C-NMR ($CDCl_3$, 175 MHz): δ = 201.3, 158.3, 134.4, 133.5, 128.7, 122.5, 114.1, 106.5, 106.4, 55.3, 50.2, 36.8, 34.0 ppm. The enantiomeric excess was determined by chiral GC on a Lipodex E column (150 ºC isotherm, 1.1 mL/min He): *R* isomer t_r = 116.11 min and *S* isomer t_r = 120.75 min.

Synthesis of compound 15d

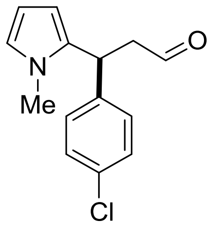

(*S*)-3-(4-Chlorophenyl)-3-(1-methyl-1*H*-pyrrol-2-yl)propanal (15d).[10] Reaction conditions and work-up were as described above, using commercially available *trans*-*p*-chloro-cinnamaldehyde (**14d**, 43.4 mg) and *N*-methylpyrrole (**11**). Purification by column chromatography on silica gel (pentane/Et_2O 4:1) gave **15d** (53.4 mg, 86%). ^{1}H-NMR ($CDCl_3$, 700 MHz): δ = 9.73 (br t, 1H), 7.28–7.26 (m, 2H), 7.11–7.10 (m, 2H), 6.58 (br t, 1H), 6.13–6.10 (m, 2H), 4.57 (t, *J* = 7.5 Hz, 1H), 3.33 (s, 3H), 3.17 (ddd, *J* = 17.4, 7.9, 1.5 Hz, 1H), 2.95 ppm (ddd, *J* = 17.3, 7.1, 1.3 Hz, 1H); ^{13}C-NMR ($CDCl_3$, 175 MHz): δ = 200.3, 141.1, 132.7, 132.5, 129.1, 129.0 122.7, 106.7, 50.0, 36.9, 33.8 ppm. The enantiomeric excess was determined by chiral GC on a Hydrodex-*β*-TBDAc column (160 ºC isotherm, 1.1 mL/min He): *S* isomer t_r = 74.69 min and *R* isomer t_r = 79.00 min. The absolute configuration was determined by comparison of the optical specific rotation of the aldehyde with reported data.[1]

Synthesis of compound 15e

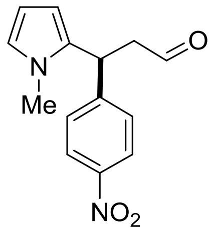

(*S*)-3-(1-Methyl-1*H*-pyrrol-2-yl)-3-(4-nitrophenyl)propanal, (15e).[10] Reaction conditions and work-up were as described above, using commercially available *trans*-*p*-nitro-cinnamaldehyde (**14e**, 44.3 mg) and *N*-methylpyrrole (**11**). Purification by column chromatography on silica gel (pentane/Et_2O 5:1) gave **15e** (64.2 mg, 99%).^{1}H-NMR ($CDCl_3$, 400 MHz): δ= 9.75 (br t, 1H), 8.13 (d, *J* = 8.7 Hz, 2H), 7.33 (d, *J* = 8.7 Hz, 2H), 6.57 (t, *J* = 2.2 Hz, 1H), 6.12 (d, *J* = 2.3 Hz, 2H), 4.69 (t, *J* = 7.3 Hz, 1H), 3.31 (s, 3H), 3.24 (ddd, *J* = 17.7, 7.5, 1.3 Hz, 1H), 3.01 ppm (ddd, *J* = 17.3, 6.9, 0.8 Hz, 1H); ^{13}C-NMR ($CDCl_3$, 125 MHz): δ= 199.6, 150.3, 146.9, 131.9, 128.9, 124.2, 123.1, 107.1, 48.8, 37.0, 34.0 ppm. The enantiomeric excess was determined by chiral GC on a Hydrodex-*β*-TBDAc column (140 °C to 200 °C, gradient 1 °C/min, 1.1 mL/min He): *S* isomer t_r = 113.36 min and *R* isomer t_r = 116.67 min. The absolute configuration was determined by comparison of the optical specific rotation of the aldehyde with reported data.[10]

5. Spectra of compounds

^{1}H and ^{13}C-NMR of compound 5

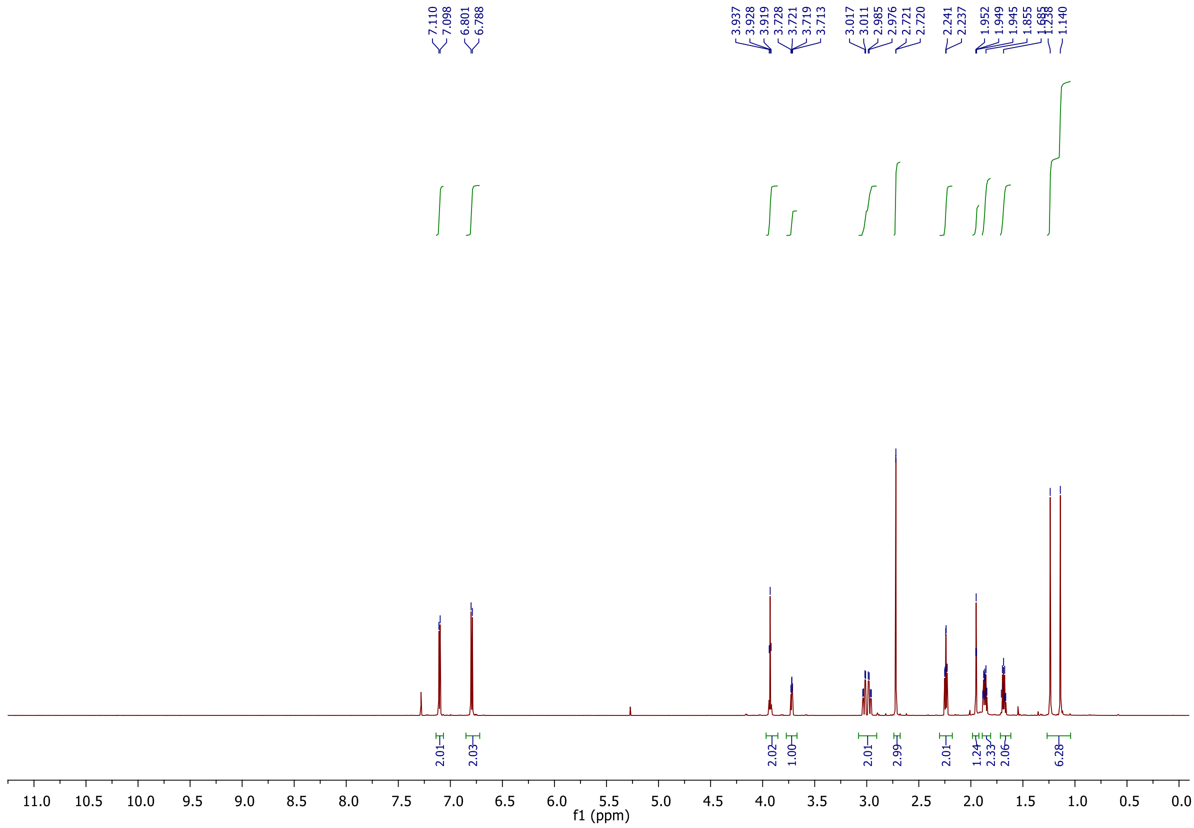

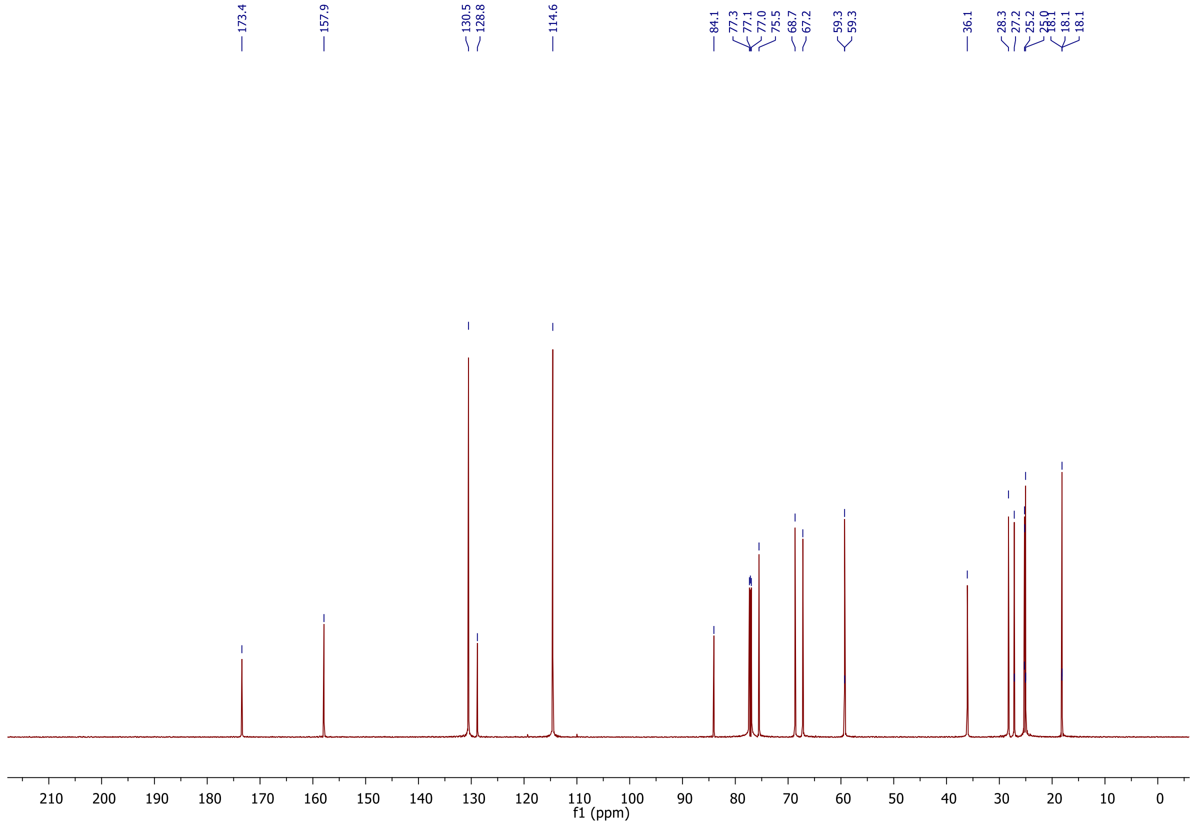

Crude ^{1}H-NMR of hPG-OMs 2a

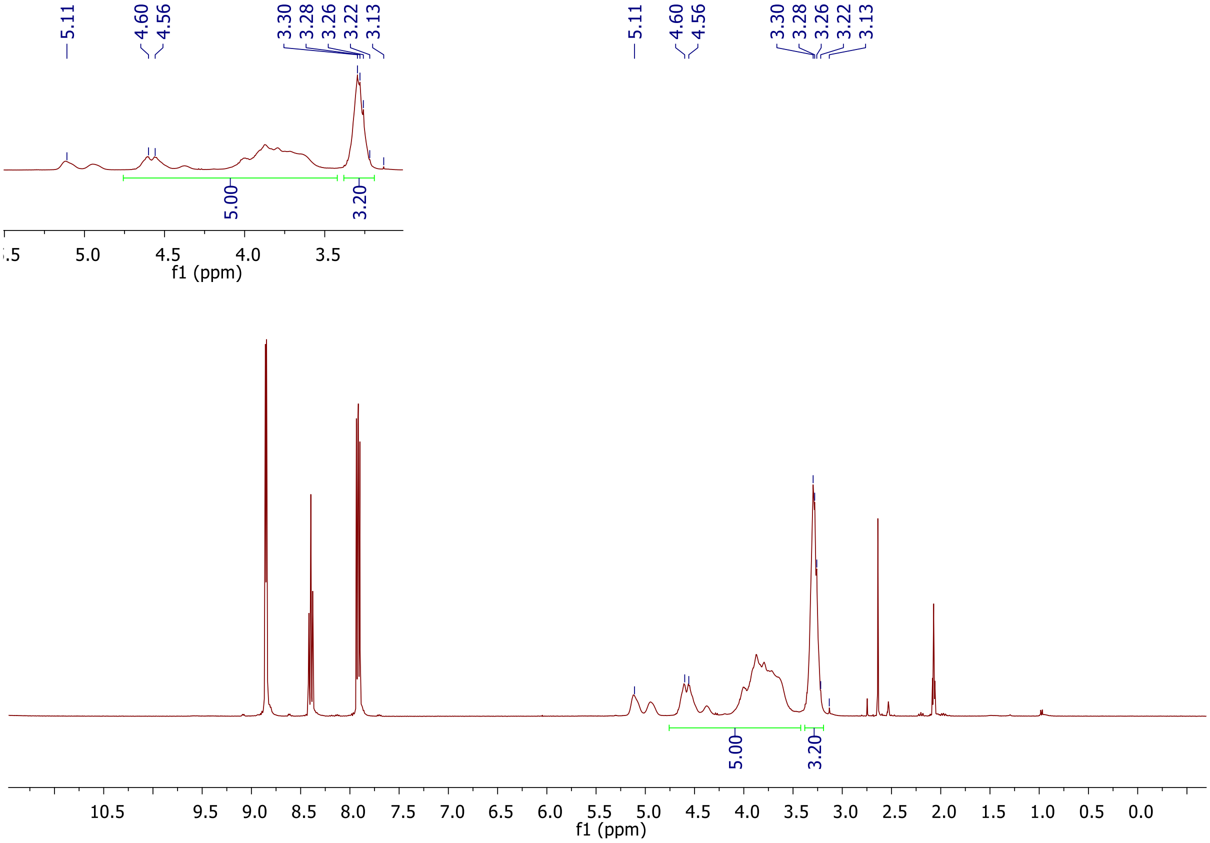

Crude ^{1}H-NMR of hPG-OMs 2b

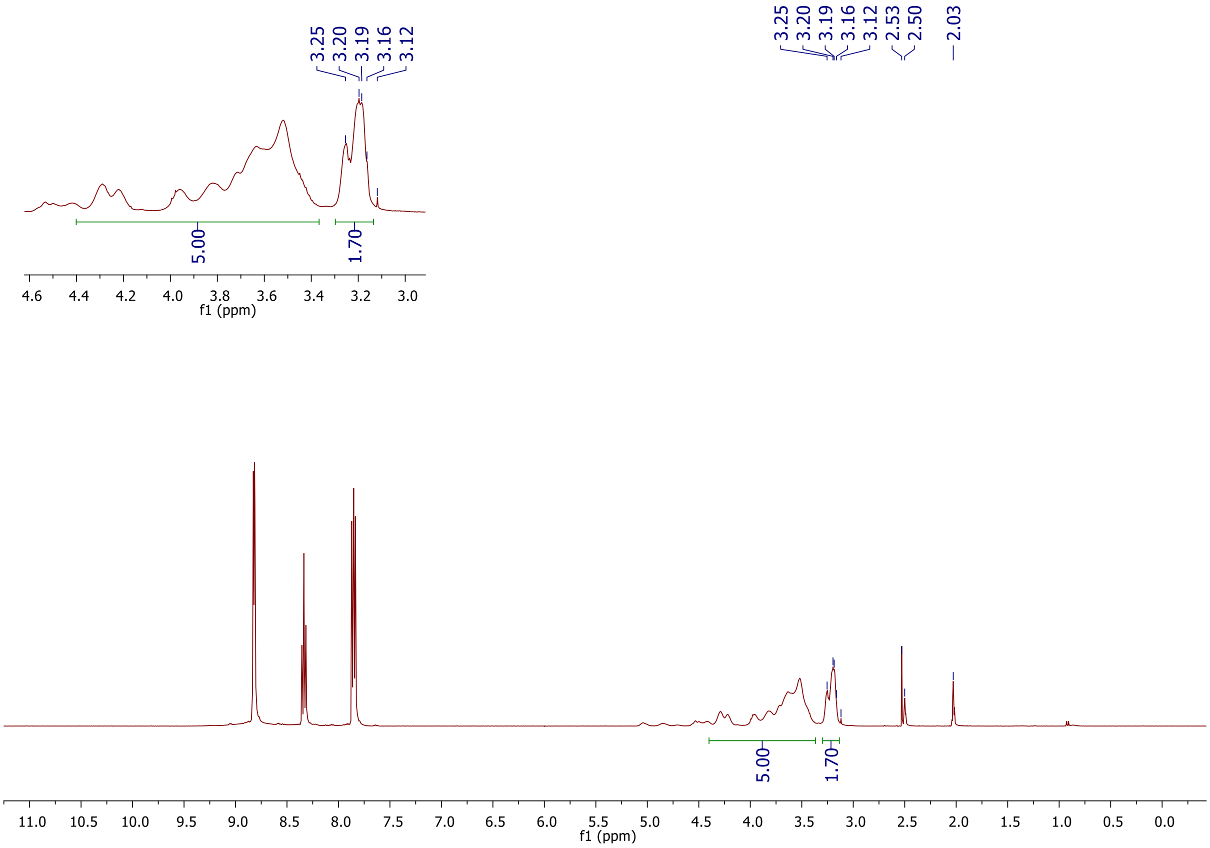

Crude ^{1}H-NMR of hPG-OMs 2c

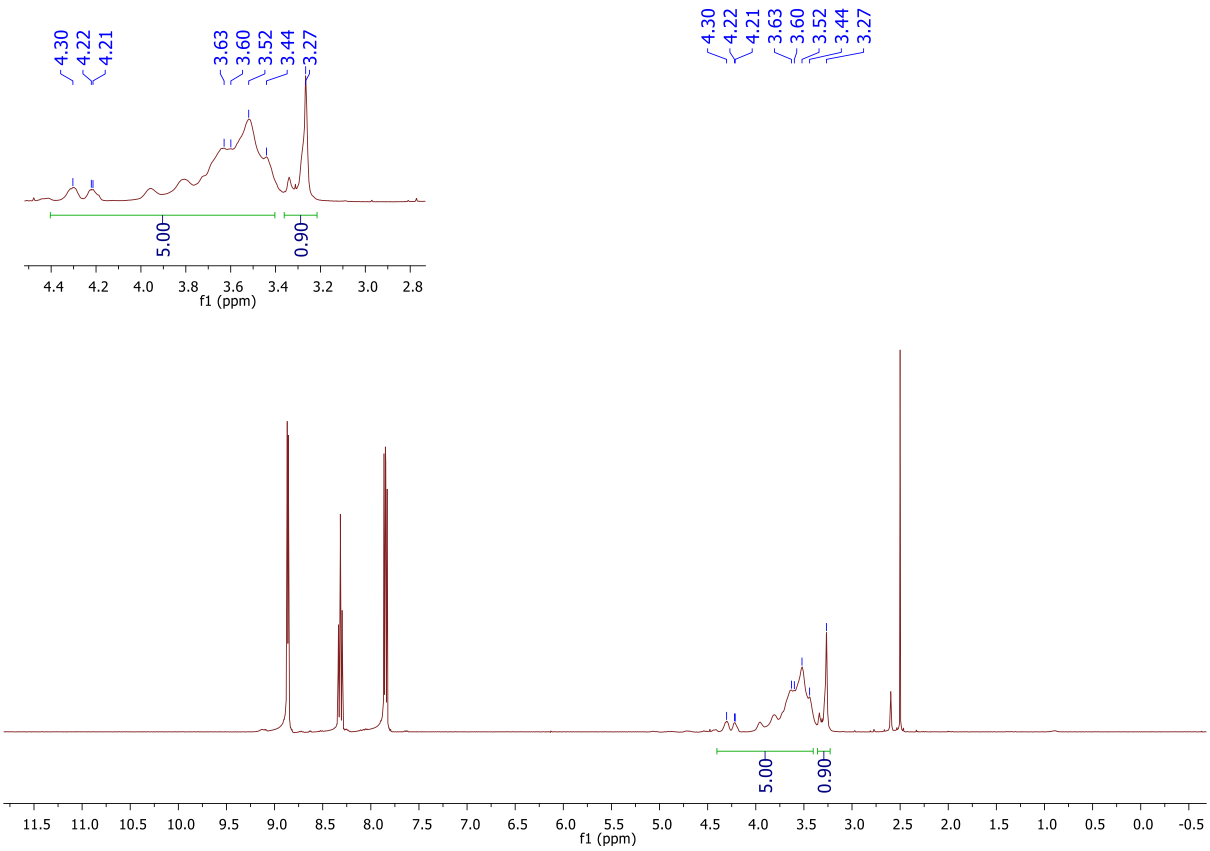

FT-IR of hPG-N_3 3a

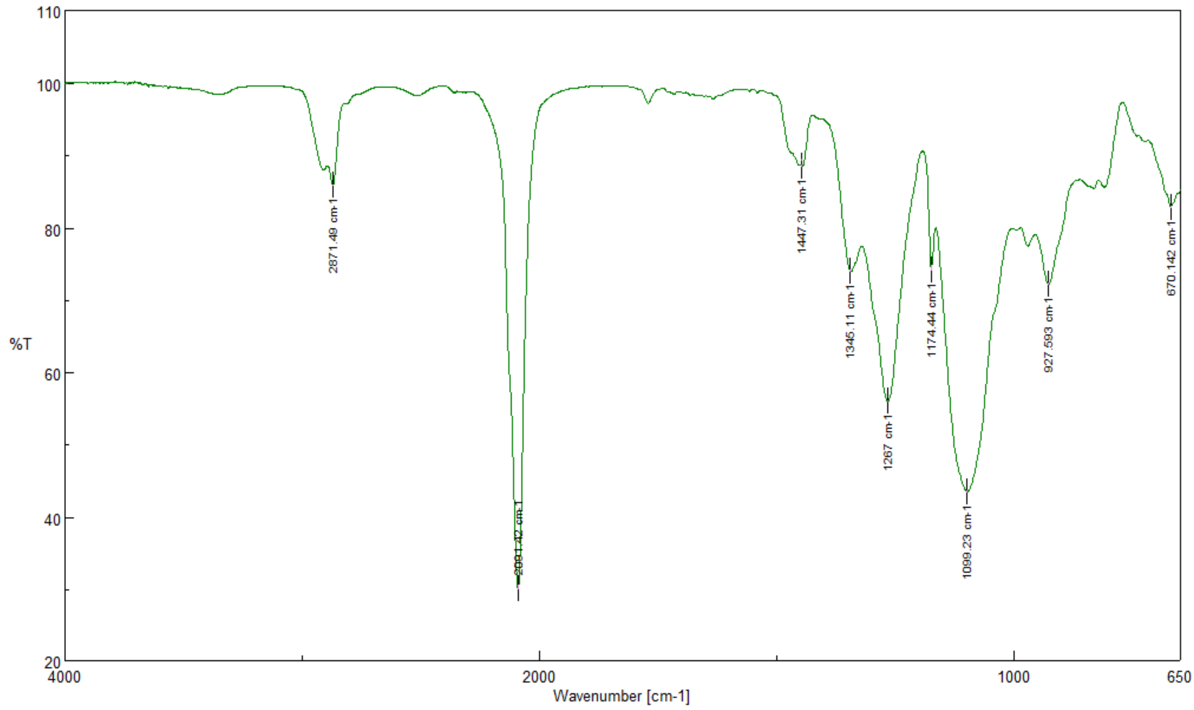

FT-IR of hPG-N_3 3b

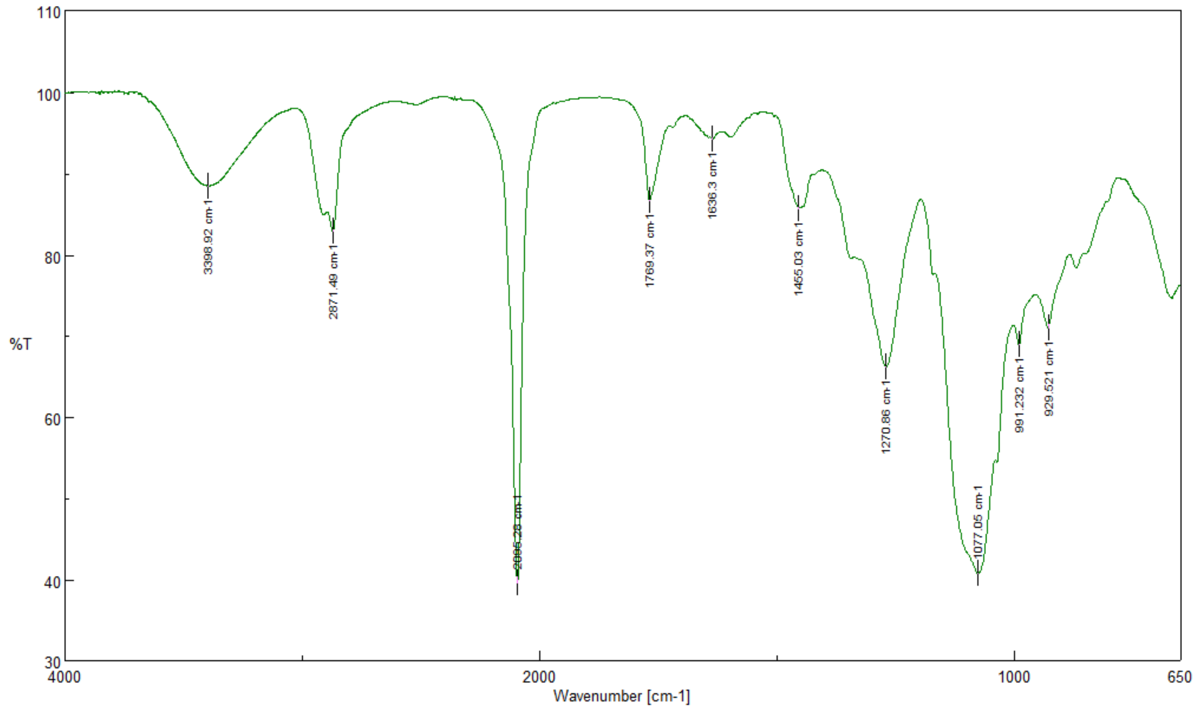

FT-IR of hPG-N_3 3c

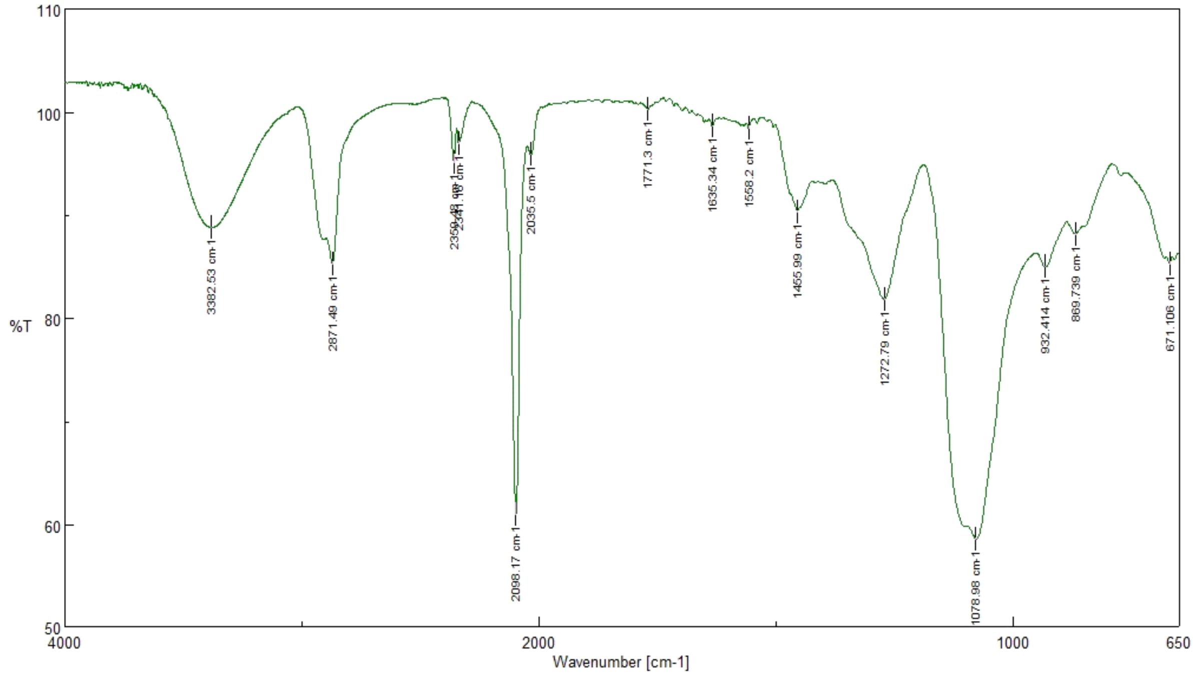

^{1}H, ^{13}C-NMR and FT-IR of compound 4a

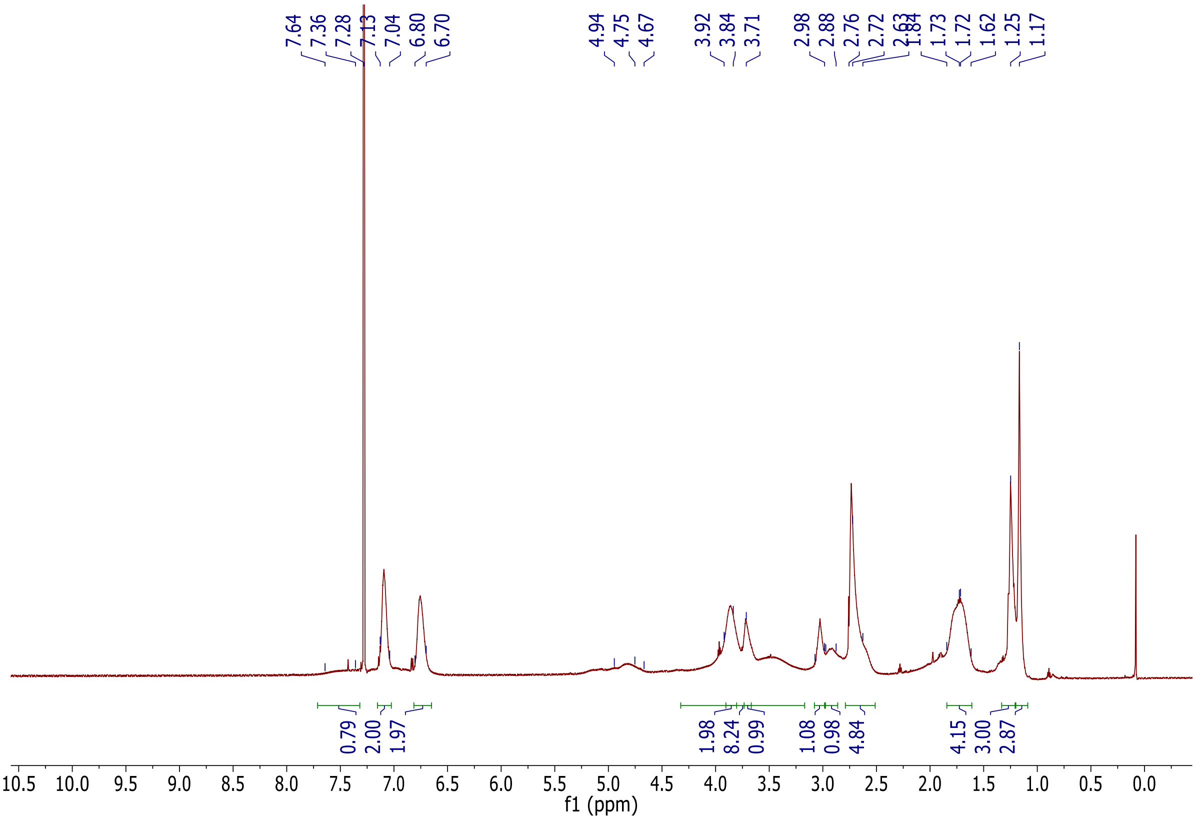

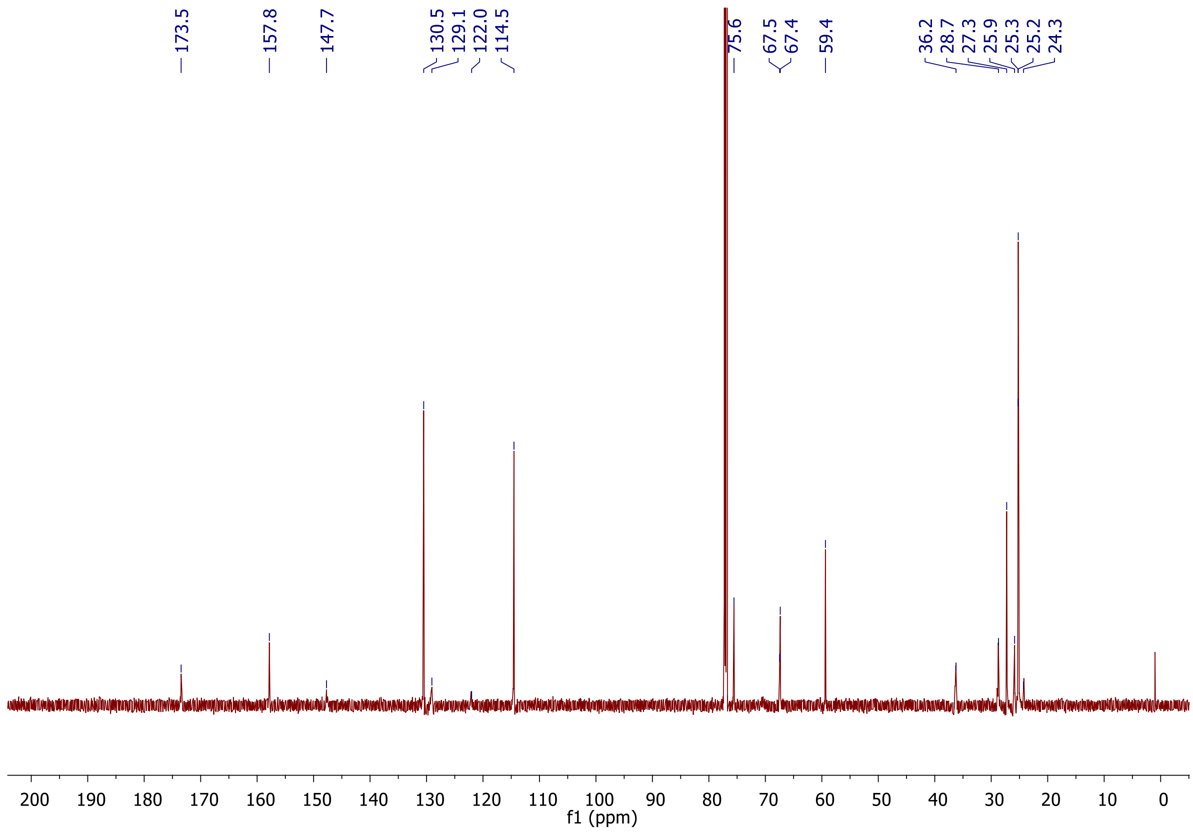

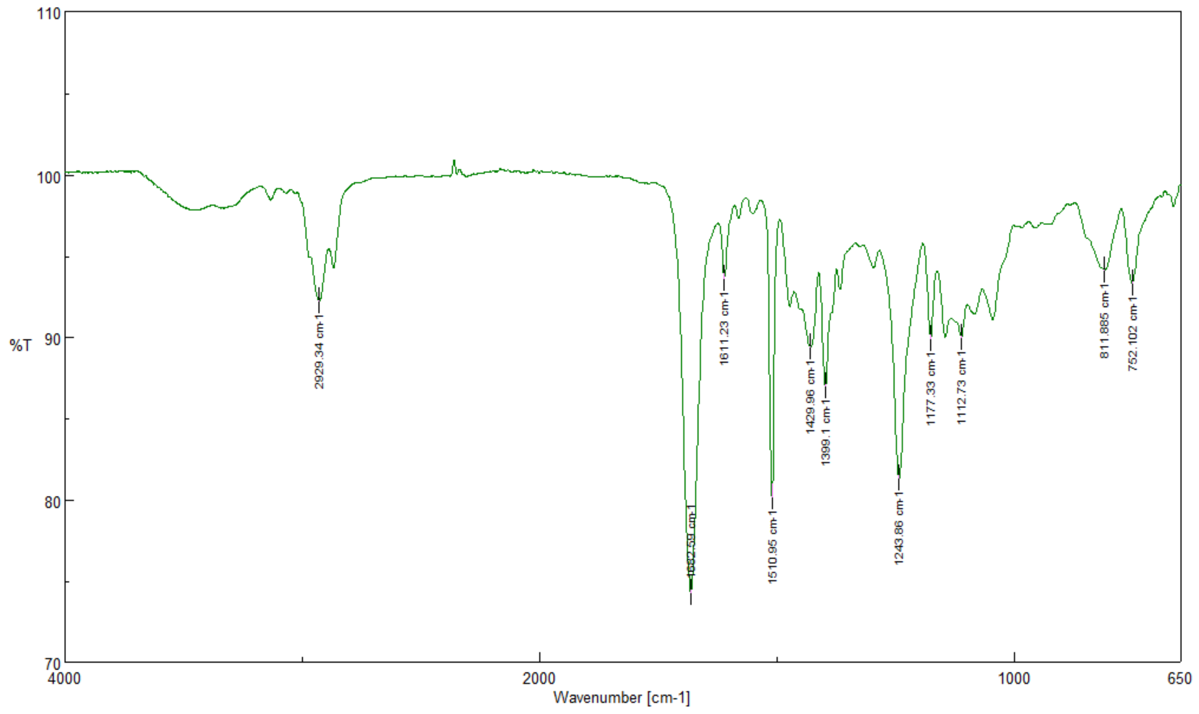

^{1}H, ^{13}C-NMR and FT-IR of compound 4b

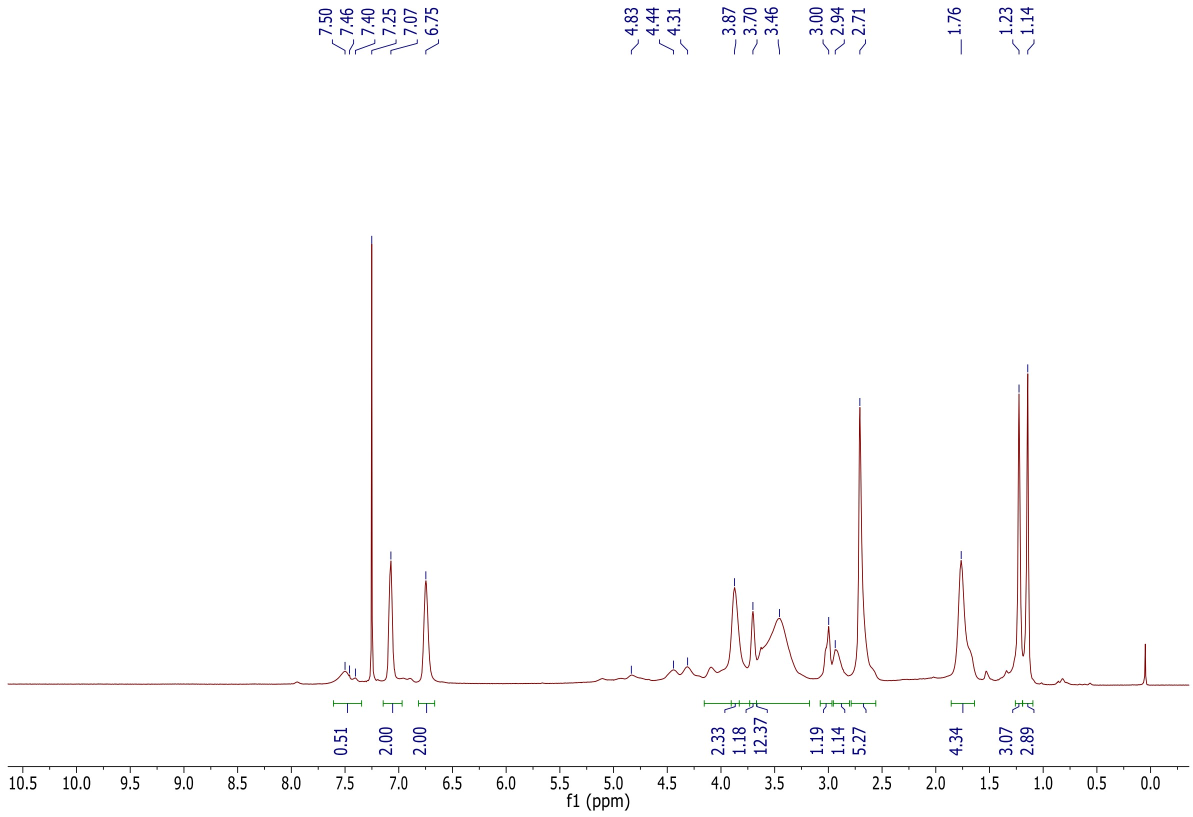

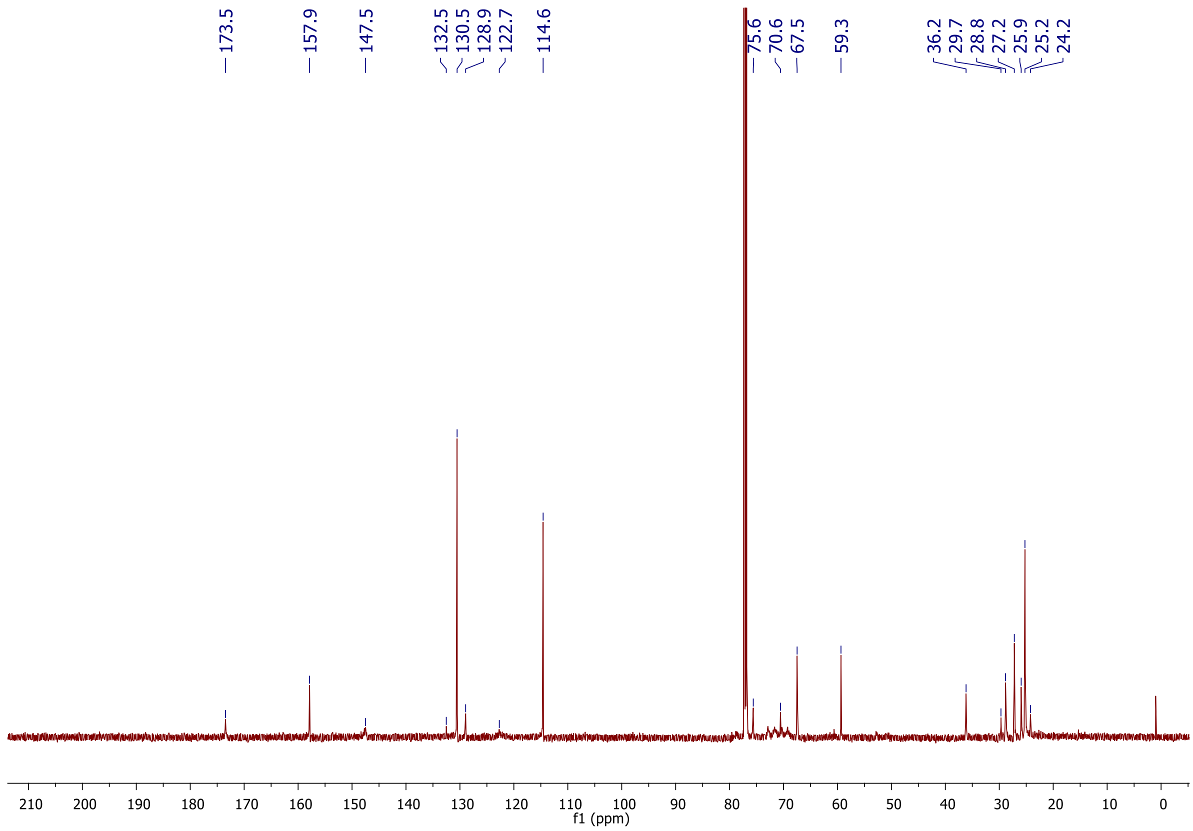
173.5
157.9
147.5
132.5
130.5
128.9
122.7
114.6
75.6
70.6
67.5
59.3
36.2
29.7
28.8
27.2
25.9
25.2
24.2
210 200 190 180 170 160 150 140 130 120 110 100 90 80 70 60 50 40 30 20 10 0
f1 (ppm)

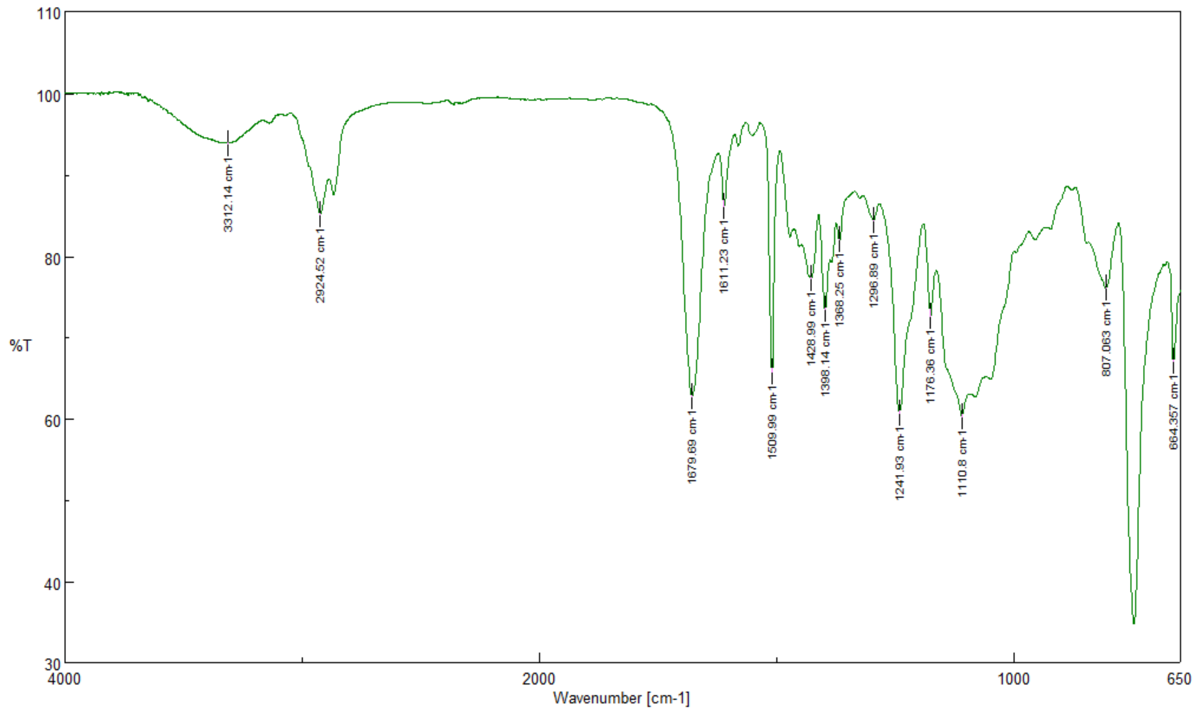
%T
110
100
80
60
40
30
4000
2000
1000
650
Wavenumber [cm-1]
3312.14 cm-1
2924.52 cm-1
1679.69 cm-1
1611.23 cm-1
1509.99 cm-1
1428.99 cm-1
1398.14 cm-1
1368.25 cm-1
1296.89 cm-1
1241.93 cm-1
1176.36 cm-1
1110.8 cm-1
807.063 cm-1
664.357 cm-1

^{1}H, ^{13}C-NMR and FT-IR of compound 4c

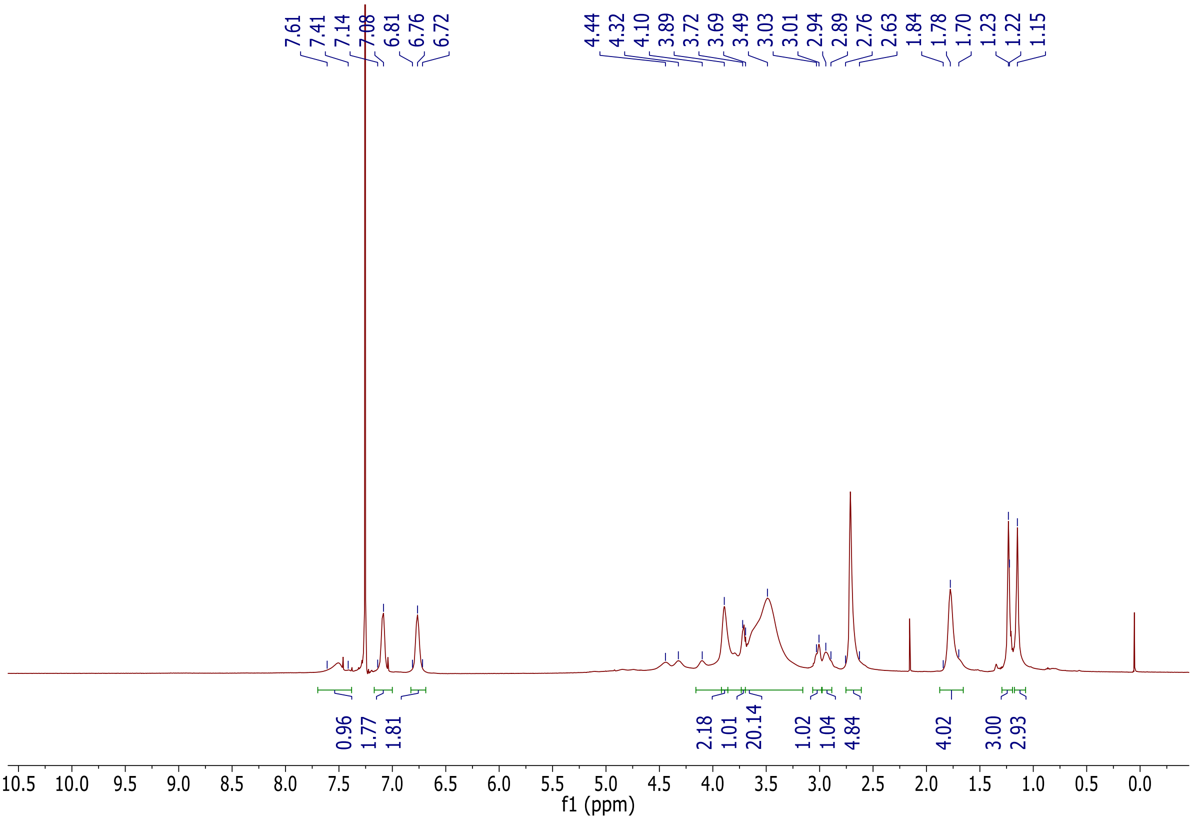

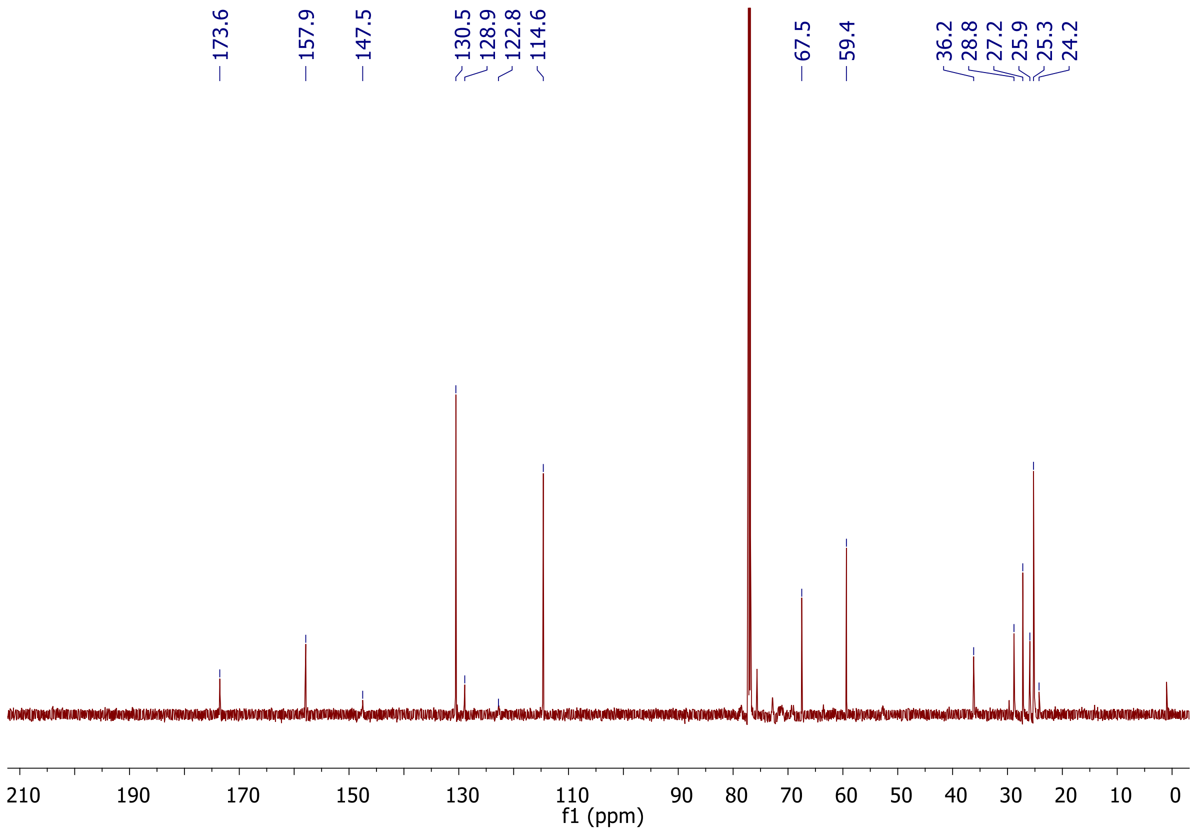

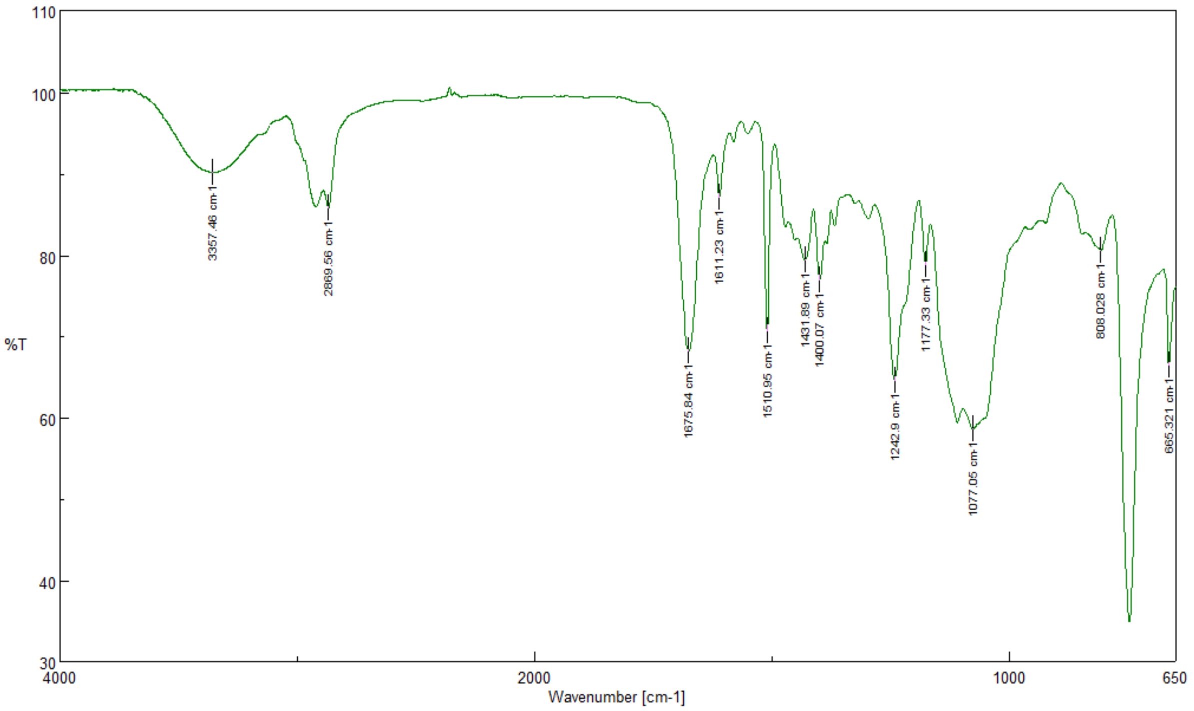

^{1}H and ^{13}C-NMR of compound 7

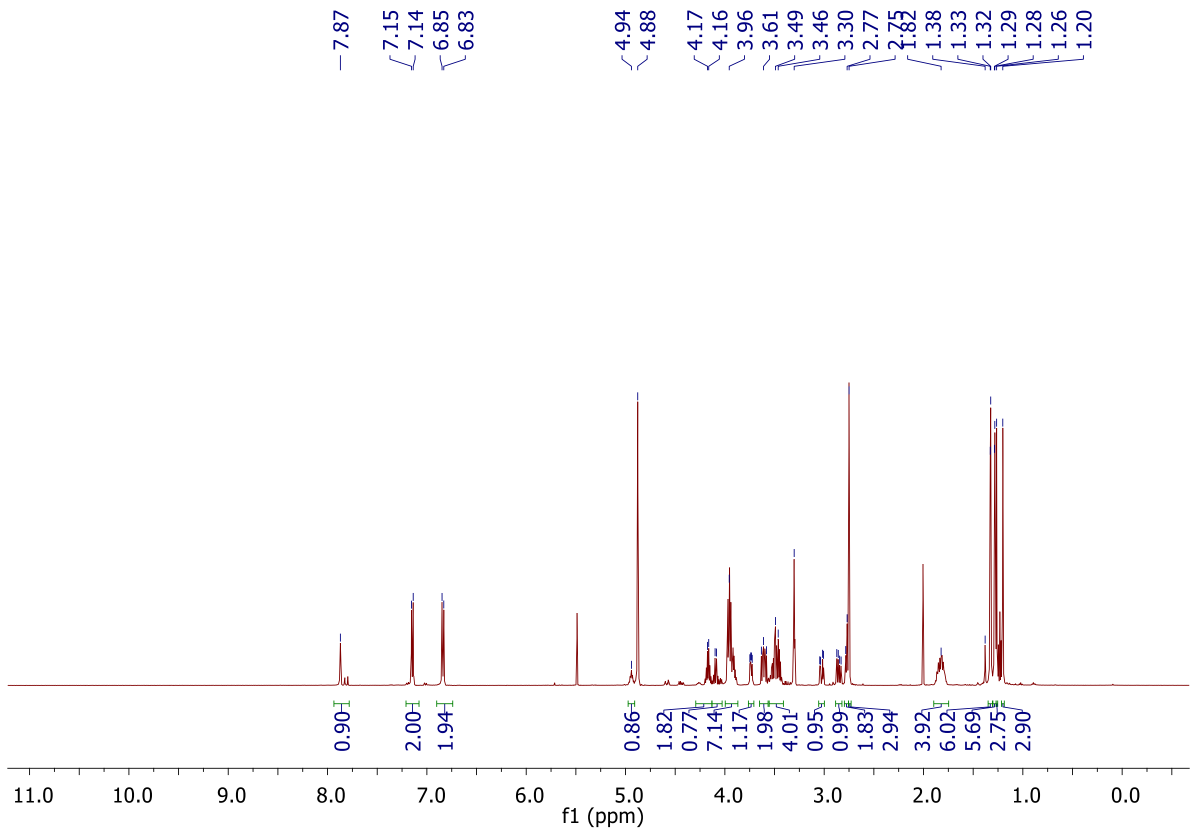

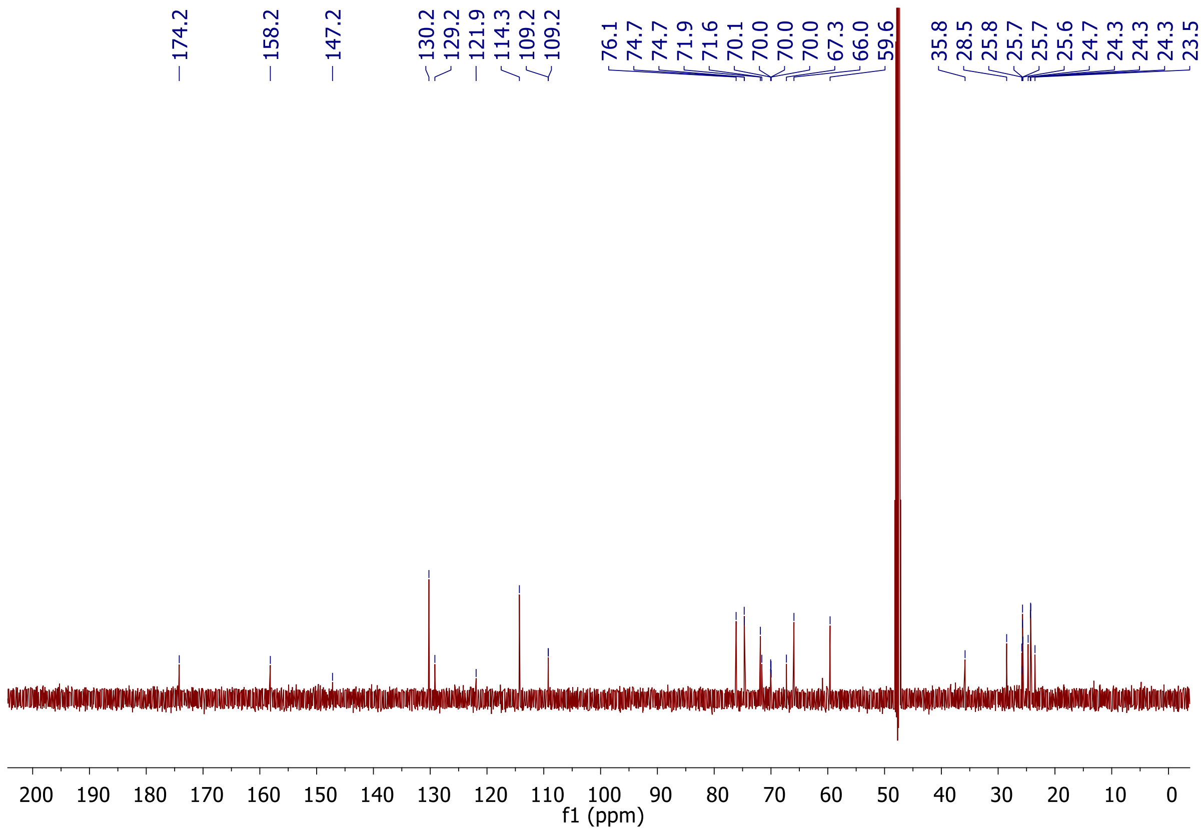

^{1}H and ^{13}C-NMR of compound 8

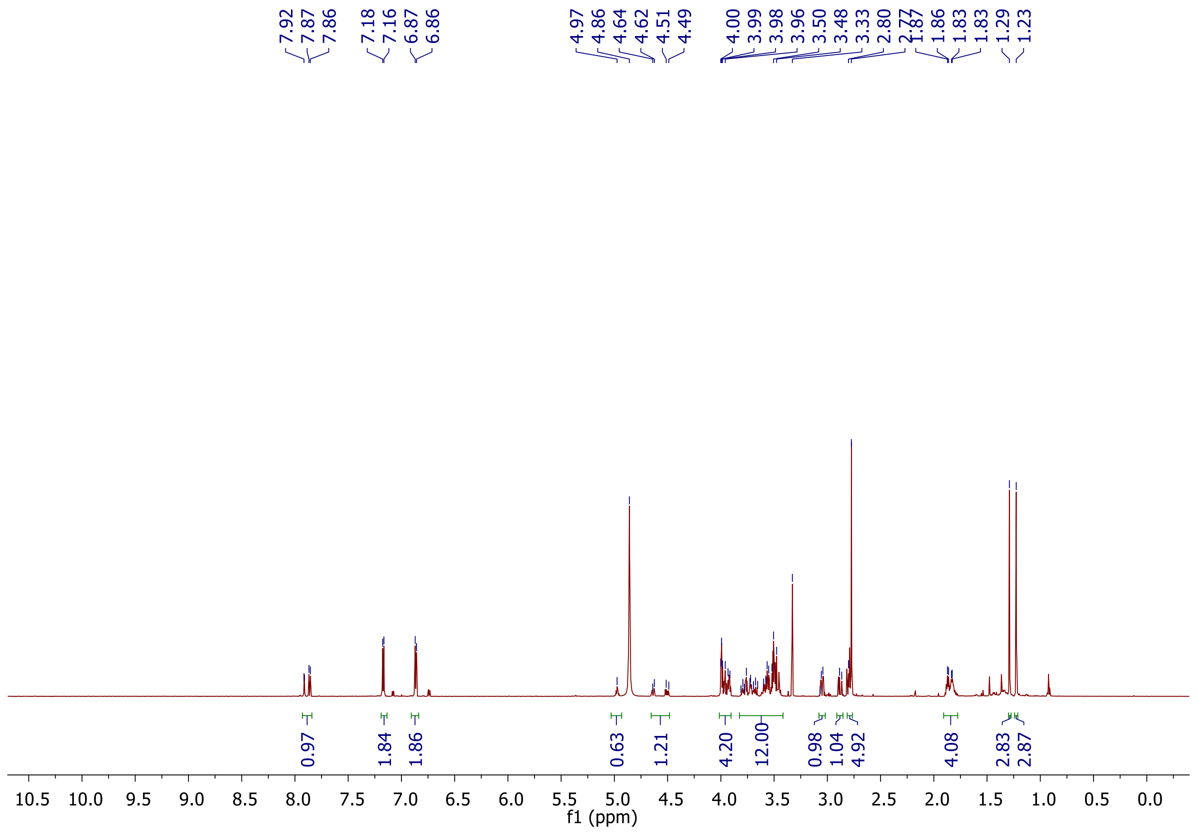

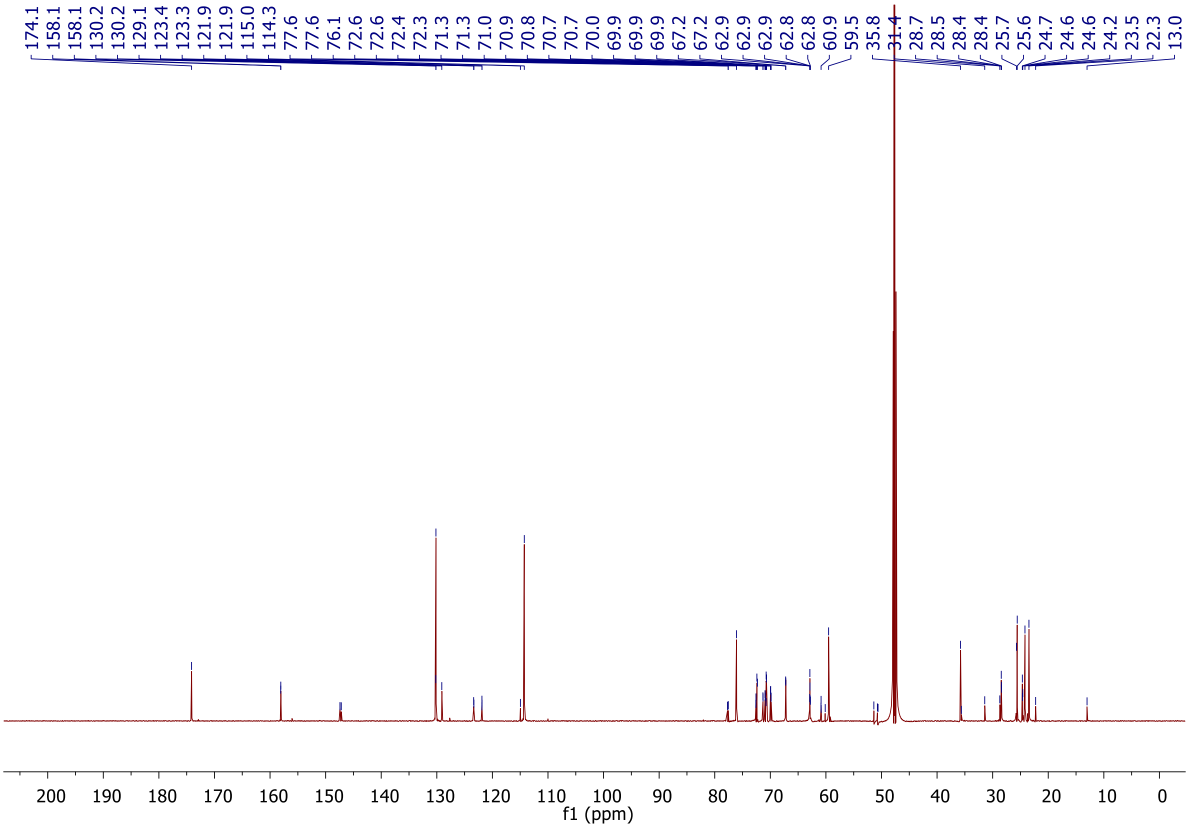
174.1
158.1
158.1
130.2
130.2
129.1
123.4
123.3
121.9
121.9
115.0
114.3
77.6
77.6
76.1
72.6
72.6
72.4
72.3
71.3
71.3
71.0
70.9
70.8
70.7
70.7
70.0
69.9
69.9
69.9
67.2
67.2
62.9
62.9
62.9
62.8
60.9
59.5
35.8
31.4
28.7
28.5
28.4
28.4
25.7
25.6
24.7
24.6
24.6
24.2
23.5
22.3
13.0
200 190 180 170 160 150 140 130 120 110 100 90 80 70 60 50 40 30 20 10 0
f1 (ppm)

Friedel–Crafts alkylations products

^{1}H and ^{13}C-NMR of compound 13

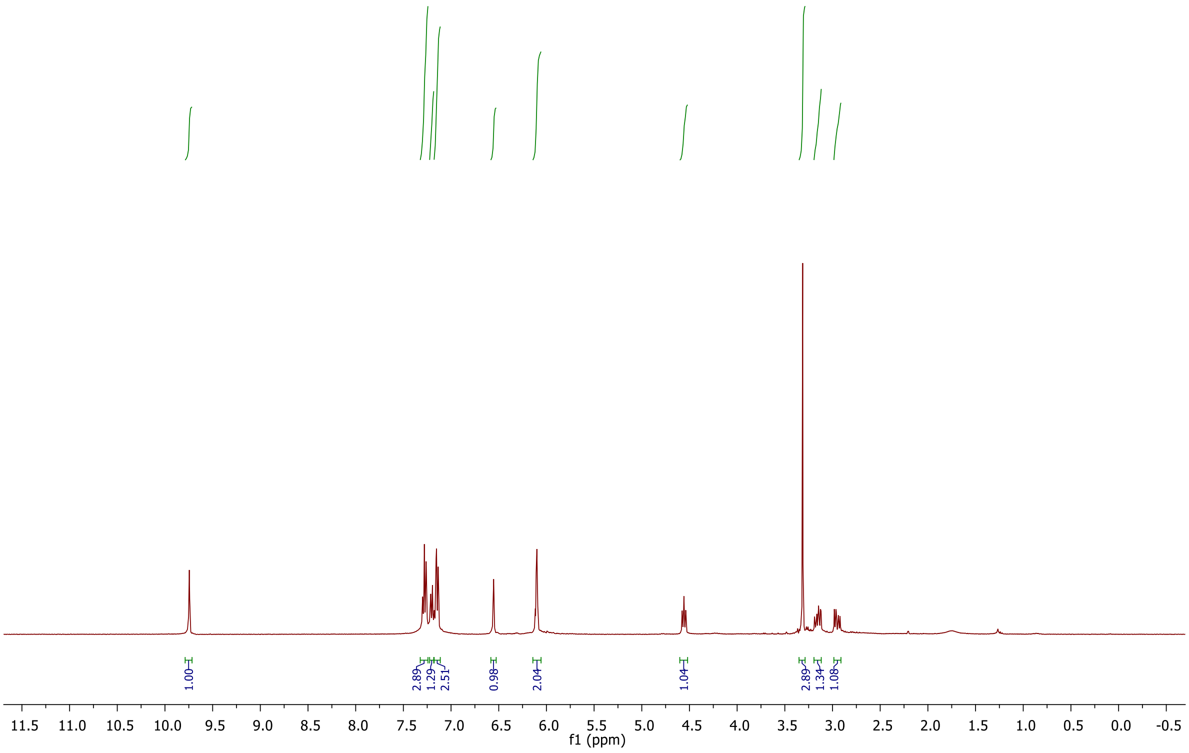

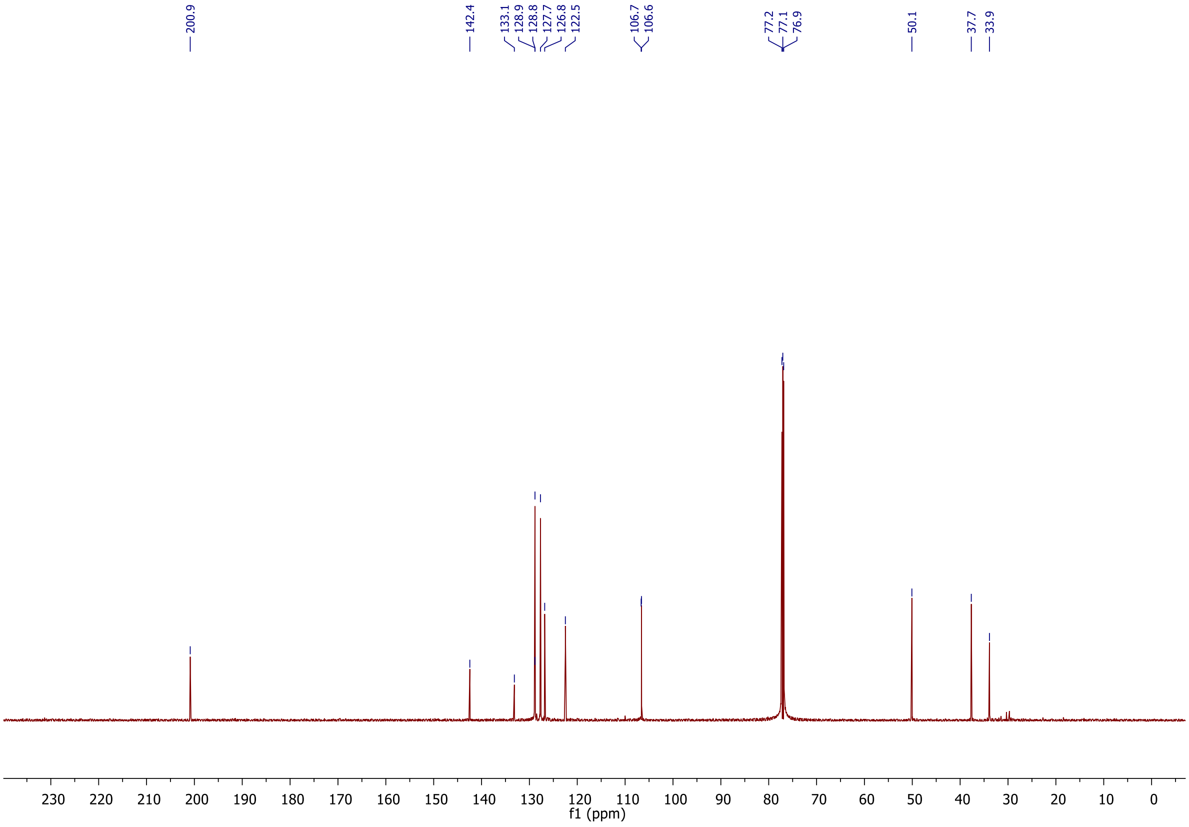

^{1}H and ^{13}C-NMR of compound 15a

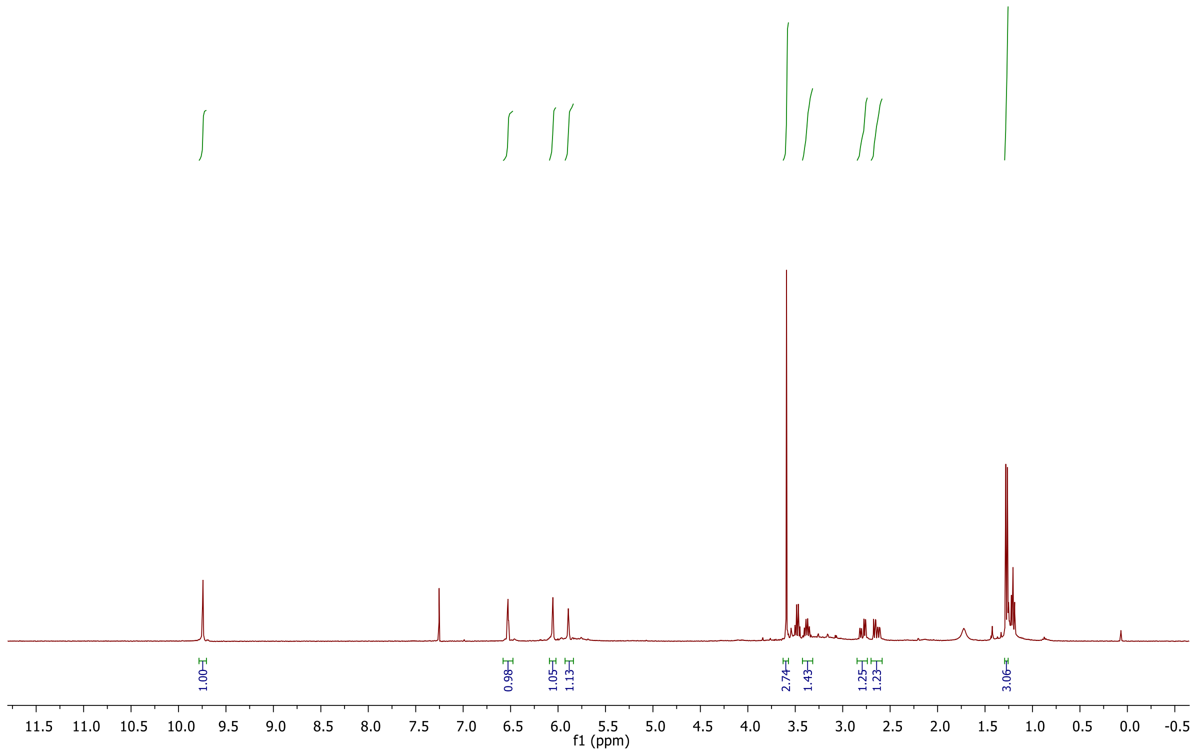

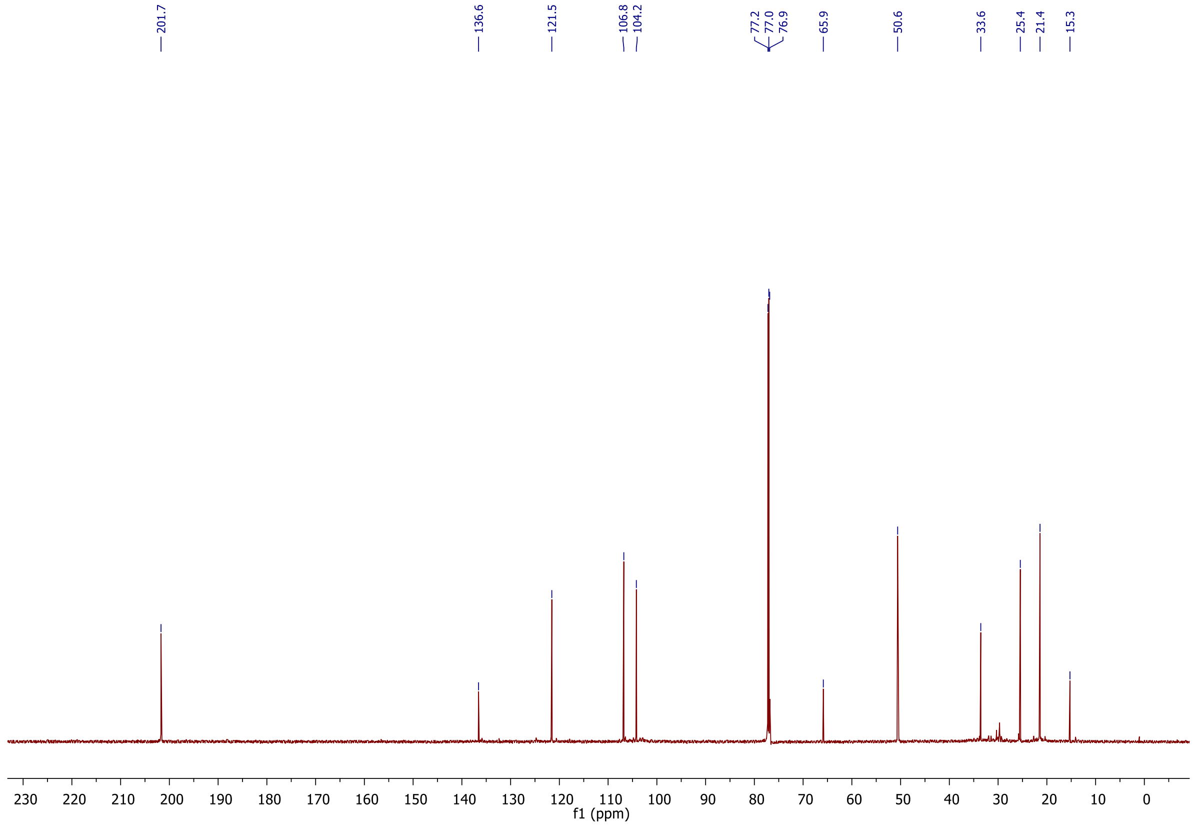

^{1}H and ^{13}C-NMR of compound 15b

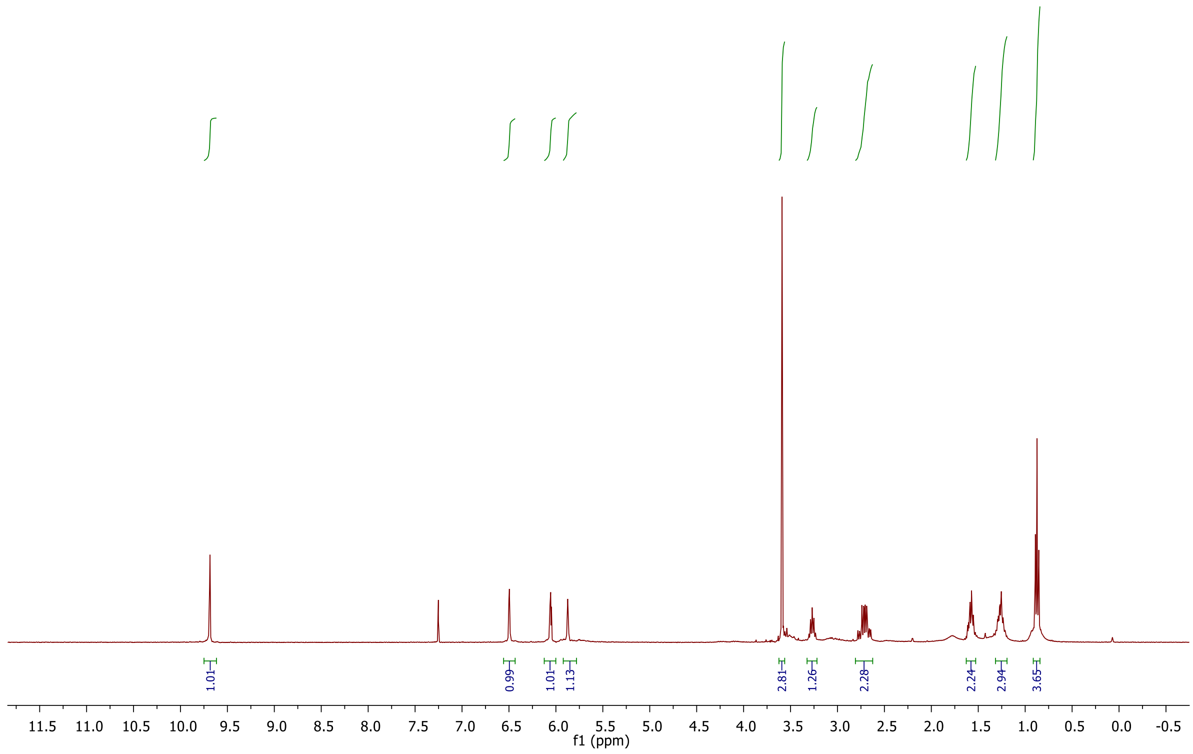

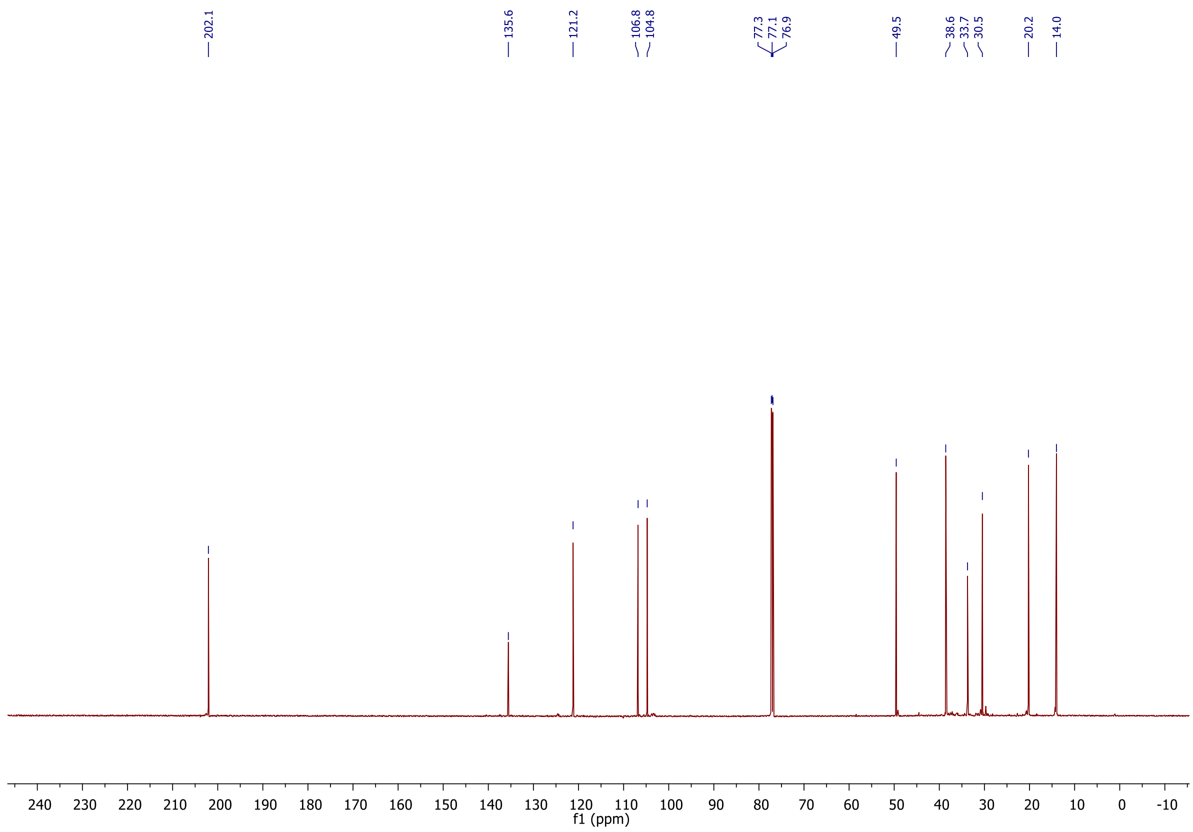

^{1}H and ^{13}C-NMR of compound 15c

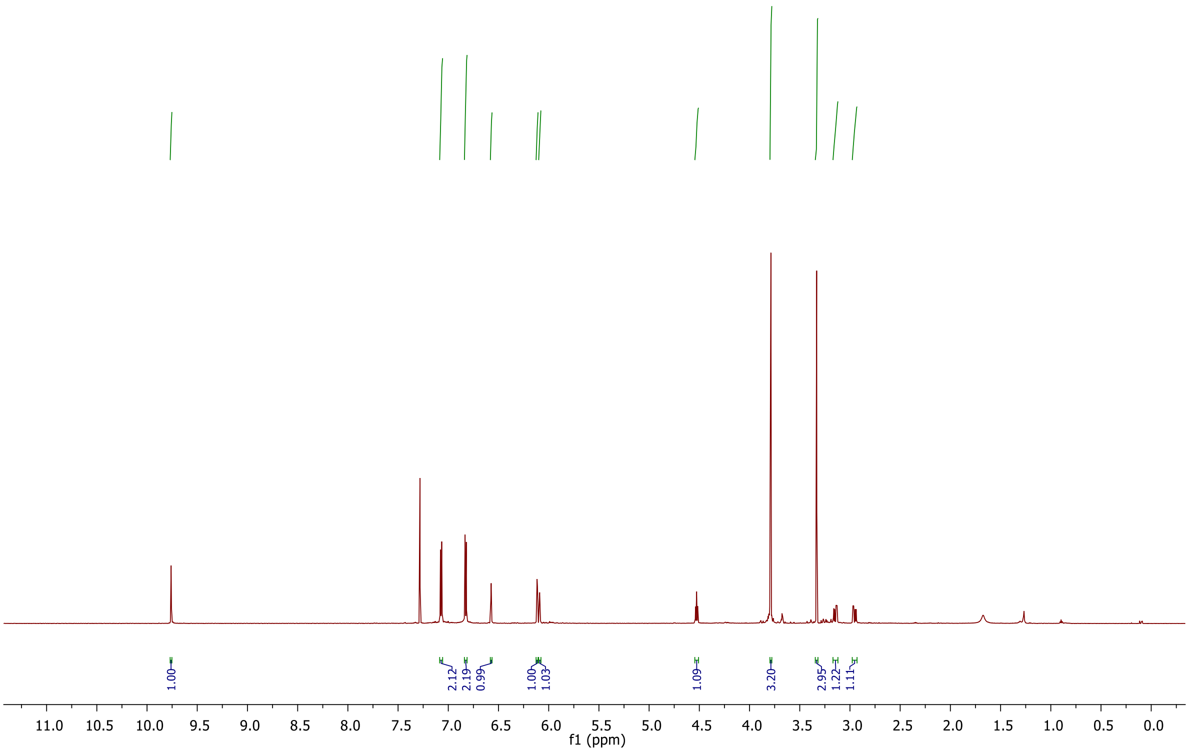

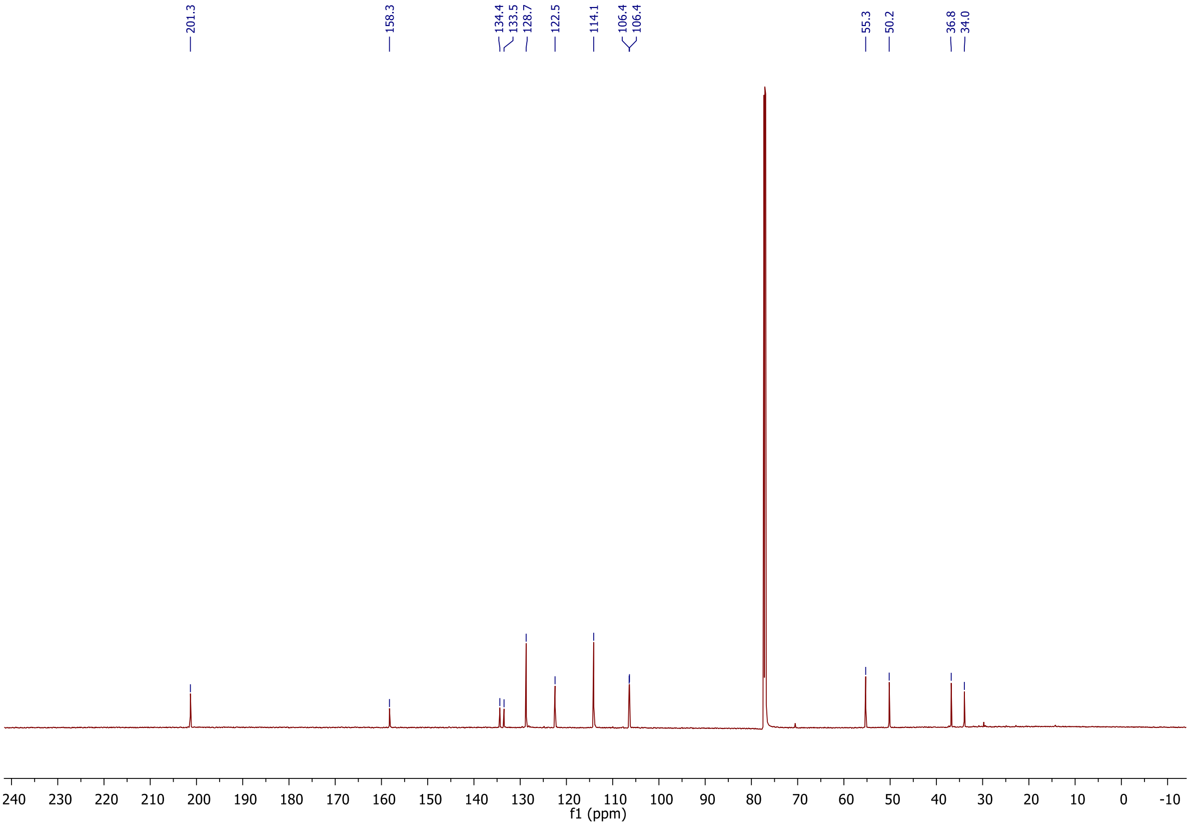

^1H and ^{13}C-NMR of compound 15d

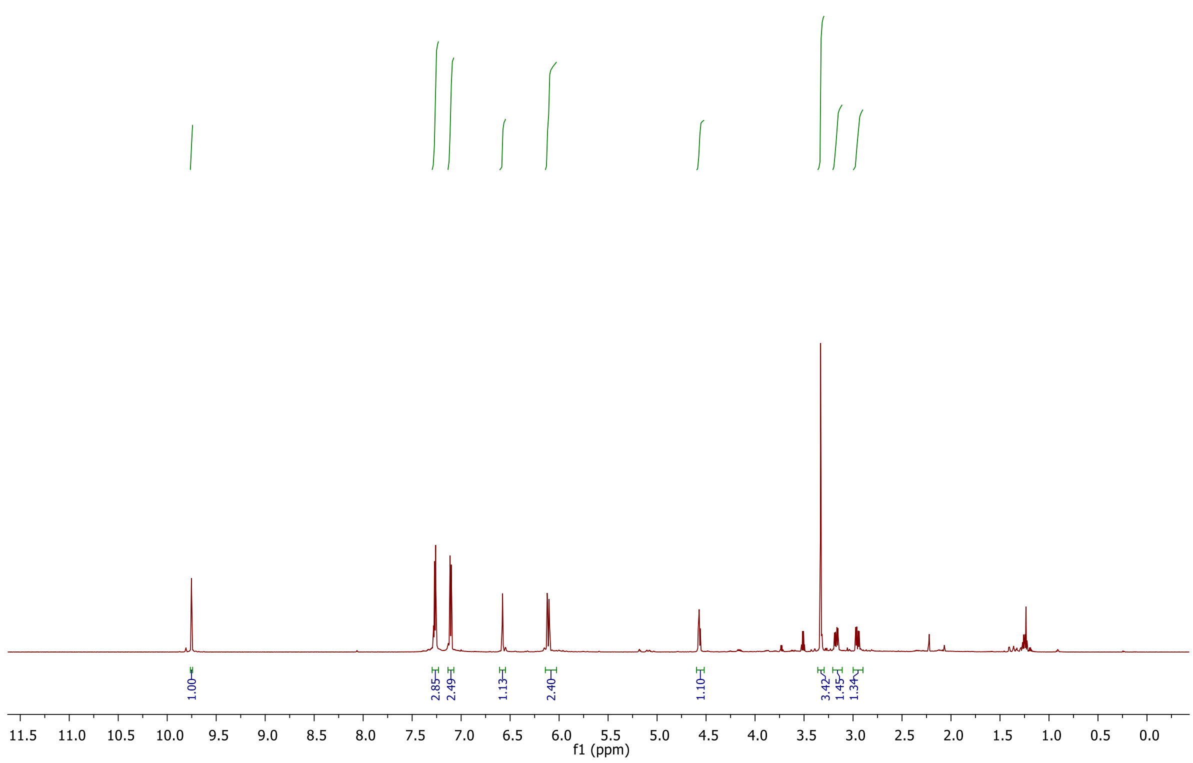

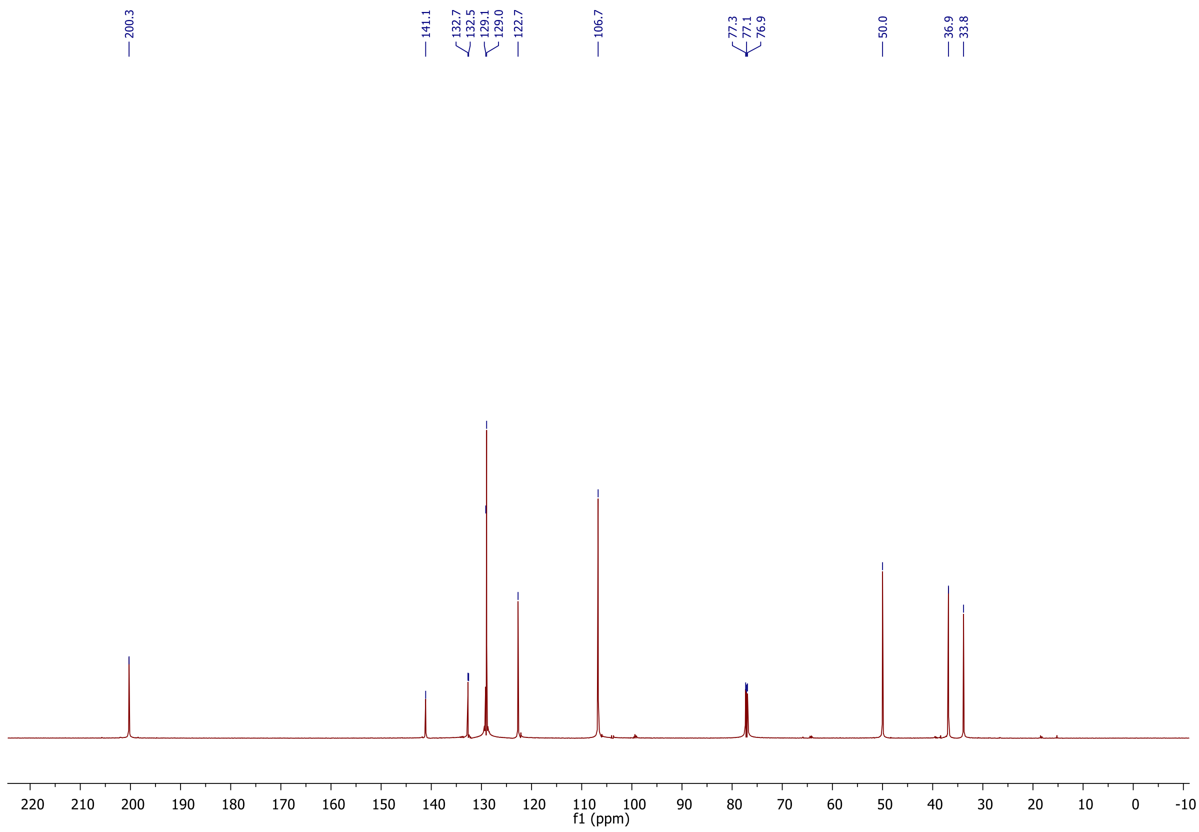

^{1}H and ^{13}C-NMR of compound 15e

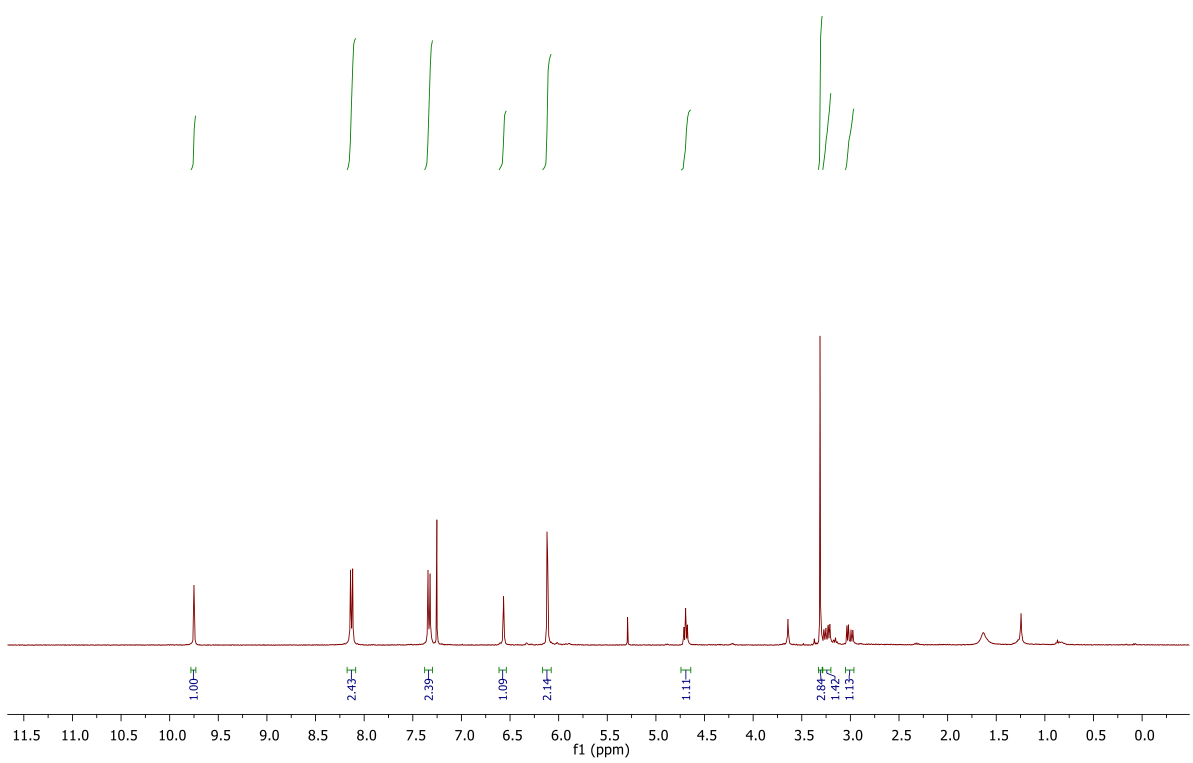

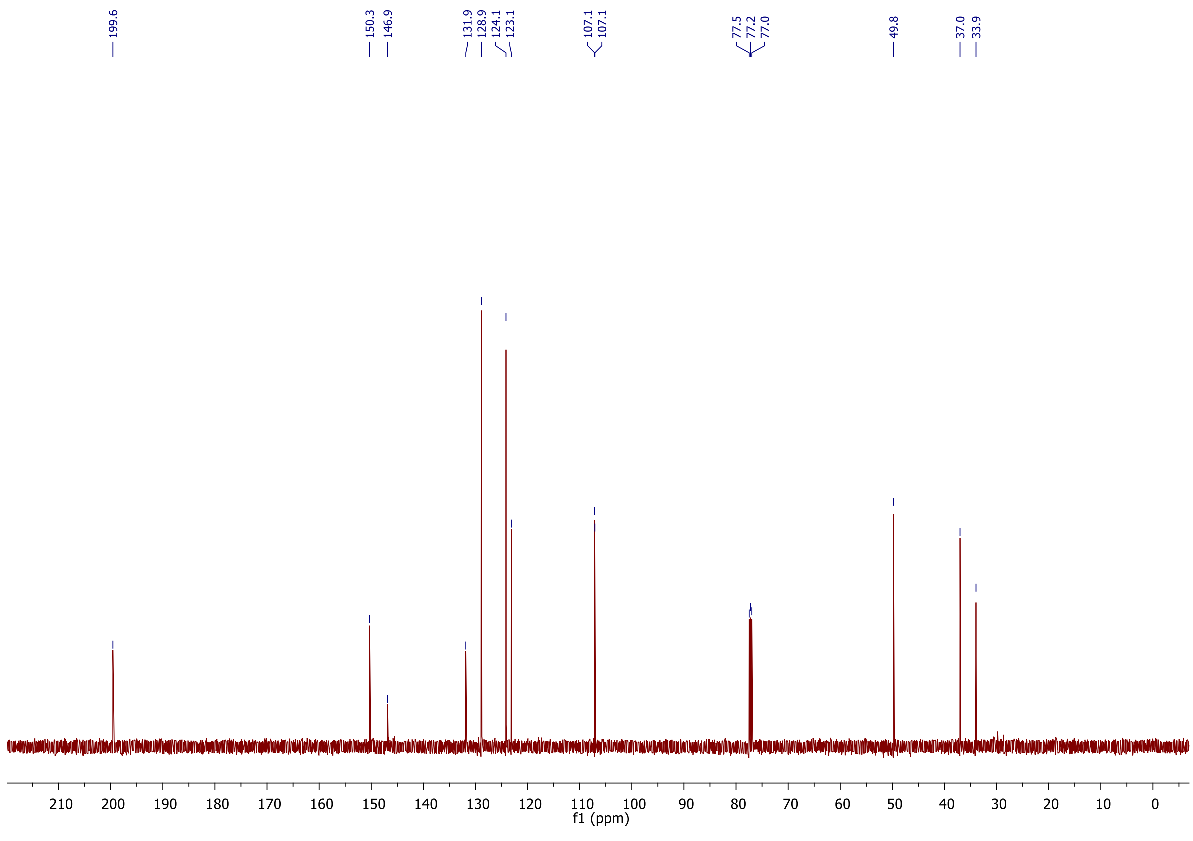

6. GC reports

Racemic 13.

```
Sample Info        : Hydrodex-ß-TBDAc
                     120 °C 200 min
                     1.1 mL/min He
                     50:1 split, 5 µL injection
                     racemic
```

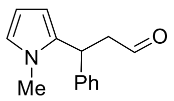

===

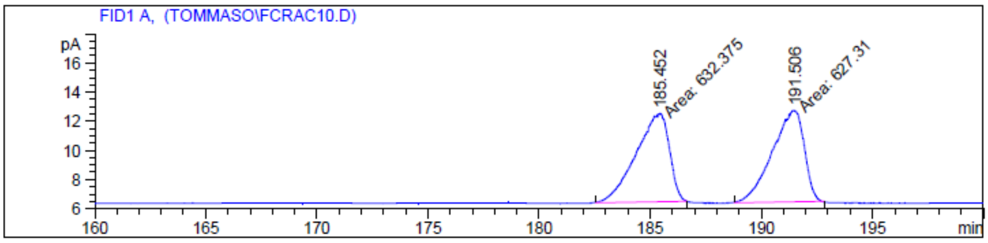

Peak #	RetTime [min]	Type	Width [min]	Area [pA*s]	Height [pA]	Area %
1	185.456	MM	1.7632	656.13544	6.20205	50.27559
2	191.506	MM	1.6850	648.94202	6.41878	49.72441

Enantioenriched 13 (optimized conditions: Table 4, entry 4).

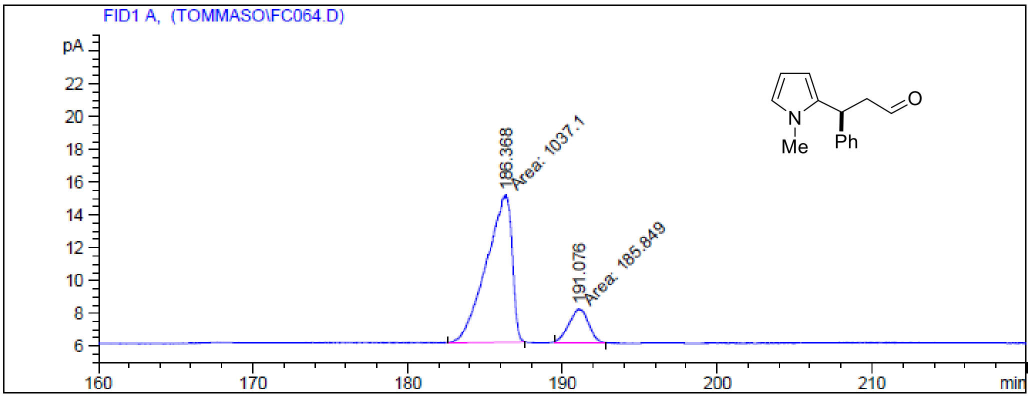

Peak #	RetTime [min]	Type	Width [min]	Area [pA*s]	Height [pA]	Area %
1	186.368	MM	1.9155	1037.09875	9.02356	84.80318
2	191.076	MM	1.4762	185.84918	2.09825	15.19682

Racemic 15a.

Sample Info : Hydrodex-ß-TBDAc
130 °C
1.1 mL/min He
50:1 split
racemic from crotonaldehyde

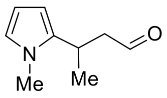

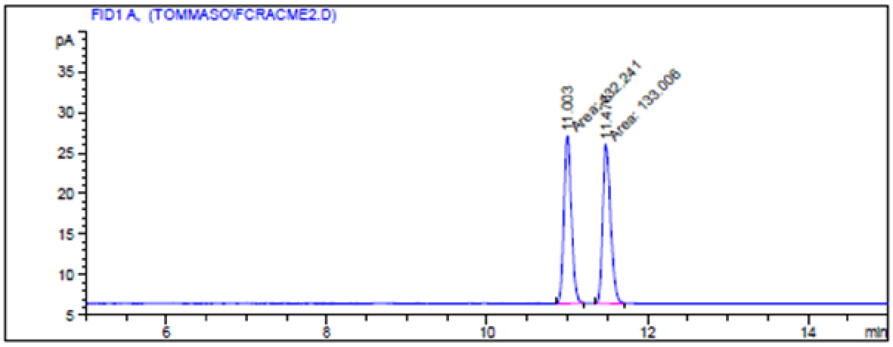

Peak #	RetTime [min]	Type	Width [min]	Area [pA*s]	Height [pA]	Area %
1	11.004	MM	0.1064	131.61288	20.62437	50.38513
2	11.475	MM	0.1107	129.60085	19.50818	49.61487

Enantioenriched 15a.

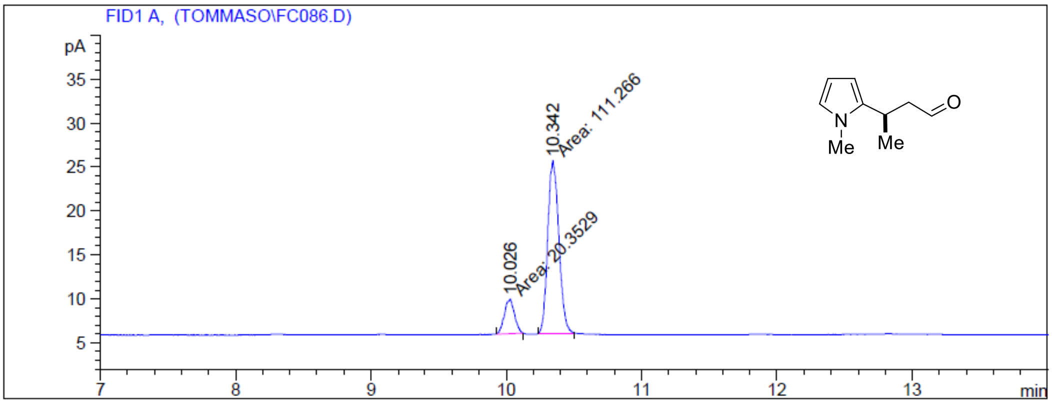

Peak #	RetTime [min]	Type	Width [min]	Area [pA*s]	Height [pA]	Area %
1	10.026	MM	0.0861	20.35293	3.93884	15.46349
2	10.342	MM	0.0946	111.26637	19.60088	84.53651

Racemic 15b.

```
Sample Info      : Hydrodex-ß-TBDAc
                   90 °C
                   1.1 mL/min He
                   50:1 split
                   racemic nPr
```

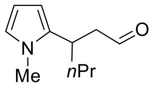

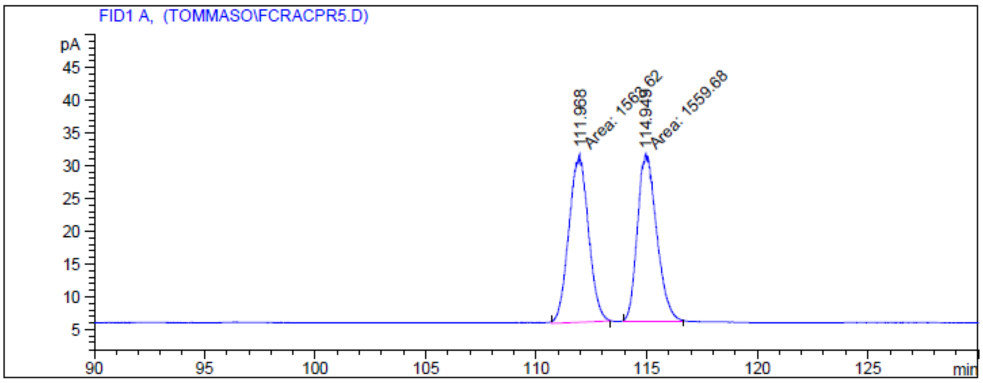

Peak #	RetTime [min]	Type	Width [min]	Area [pA*s]	Height [pA]	Area %
1	111.968	MM	1.0052	1563.62000	25.92435	50.06300
2	114.949	MM	1.0048	1559.68433	25.86973	49.93700

Enantioenriched 15b.

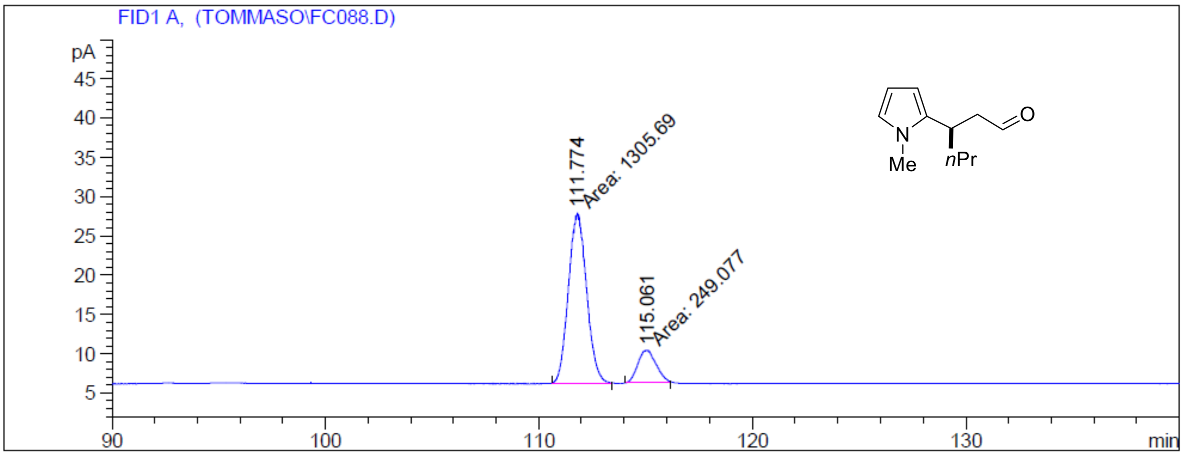

Peak #	RetTime [min]	Type	Width [min]	Area [pA*s]	Height [pA]	Area %
1	111.774	MM	0.9970	1305.69275	21.82700	83.97980
2	115.061	MM	0.9835	249.07718	4.22106	16.02020

Racemic 15c.

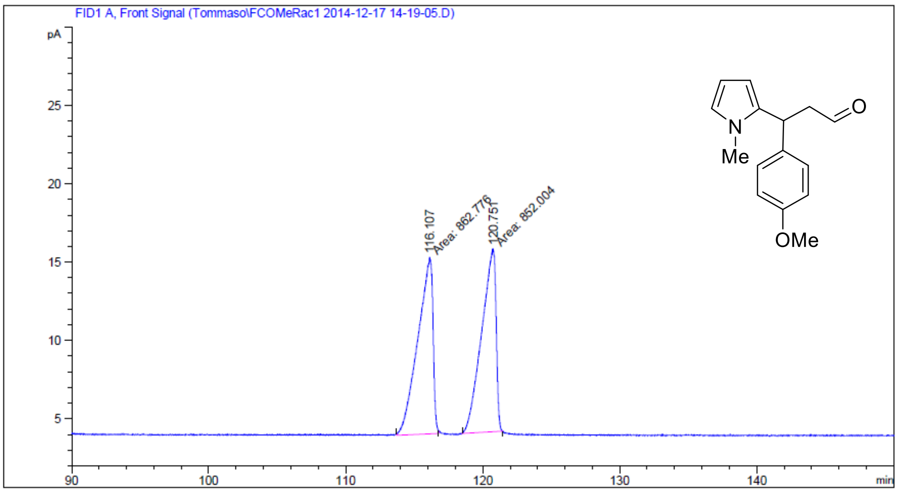

Peak #	RetTime [min]	Type	Width [min]	Area [pA*s]	Area %	Name
3	4.973		0.0000	0.00000	0.00000	C15
4	5.556		0.0000	0.00000	0.00000	C16
5	116.107	MM	1.2765	862.77594	50.31410	?
6	120.751	MM	1.2152	852.00378	49.68590	?

Enantioenriched 15c.

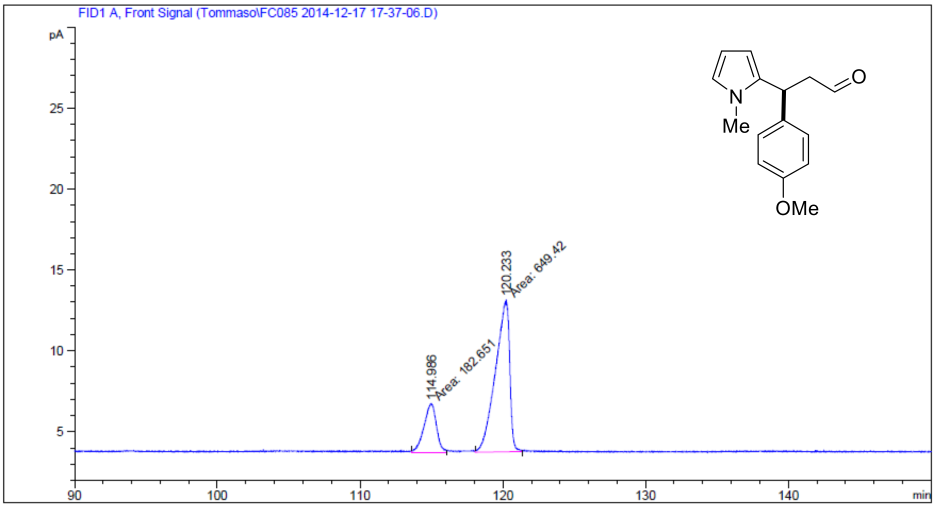

Peak #	RetTime [min]	Type	Width [min]	Area [pA*s]	Area %	Name
3	4.973		0.0000	0.00000	0.00000	C15
4	5.556		0.0000	0.00000	0.00000	C16
5	114.986	MM	1.0073	182.65097	21.95136	?
6	120.233	MM	1.1532	649.42023	78.04864	?

Racemic 15d.

Sample Info : Hydrodex-ß-TBDAc
160 °C
1.1 mL/min He
50:1 split
racemic

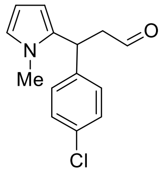

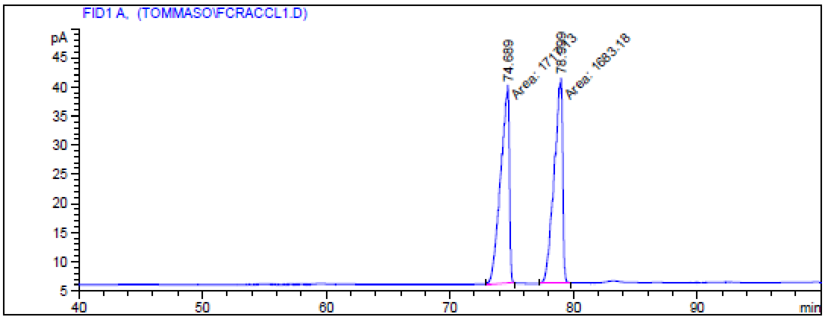

Peak #	RetTime [min]	Type	Width [min]	Area [pA*s]	Height [pA]	Area %
1	74.689	MM	0.8409	1714.01038	33.97167	50.14686
2	78.999	MM	0.7999	1703.97095	35.50229	49.85314

Enantioenriched 15d.

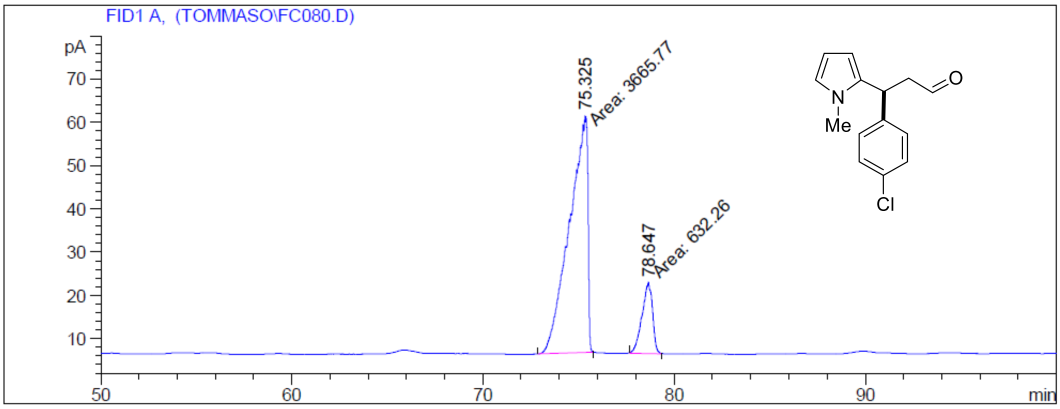

Peak #	RetTime [min]	Type	Width [min]	Area [pA*s]	Height [pA]	Area %
1	75.325	MM	1.1146	3665.77295	54.81347	85.28955
2	78.647	MM	0.6337	632.25977	16.62761	14.71045

Racemic 15e.

Sample Info : Hydrodex-ß-TBDAc
140 °C to 200 °C, 1 °C/min
1.1 mL/min He
50:1 split
racemic

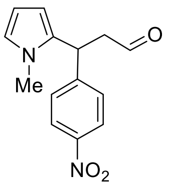

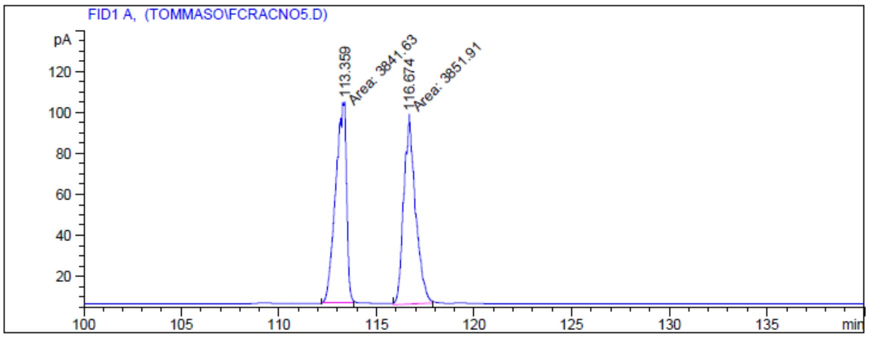

Peak #	RetTime [min]	Type	Width [min]	Area [pA*s]	Height [pA]	Area %
1	113.359	MM	0.6509	3841.62915	98.36903	49.93319
2	116.674	MM	0.6930	3851.90869	92.63337	50.06681

Enantioenriched 15e.

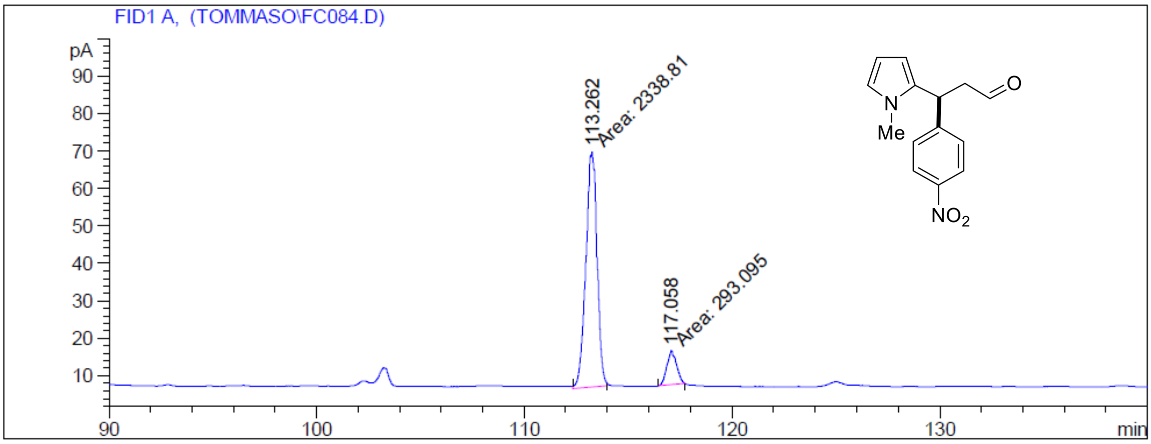

Peak #	RetTime [min]	Type	Width [min]	Area [pA*s]	Height [pA]	Area %
1	0.165	BV	0.1011	7.02994e-1	9.20222e-2	0.02670
2	113.262	MM	0.6214	2338.81006	62.72633	88.84003
3	117.058	MM	0.5343	293.09525	9.14316	11.13326

7. References

1) Zhang, Y.; Zhao, L.; Lee, S. S.; Ying, J. Y. *Adv. Synth. Catal.* **2006**, *348*, 2027–2032.

   doi: 10.1002/adsc.200600240

2) Sunder, A.; Mülhaupt, R.; Haag, R.; Frey, H. *Adv. Mater.* **2000**, *12*, 235–239.

   doi: 10.1002/(SICI)1521-4095(200002)12:3<235::AID-ADMA235>3.0.CO;2-Y

3) Roller, S.; Zhou, H; Haag, R. *Molec. Divers.* **2005**, *9*, 305–316.

   doi: 10.1007/s11030-005-8117-y

4) Paez, J. I.; Brunetti, V.; Strumia, M. C.; Becherer, T.; Solomun, T.; Miguel, J.; Hermanns, C. F.; Calderón, M.; Haag, R. *J. Mater. Chem.* **2012**, *22*, 19488–19497.

   doi: 10.1039/c2jm32486e

5) Rostovtsev, V. V.; Green, L. G.; Fokin, V. V.; K. B. Sharpless, *Angew. Chem. Int. Ed.* **2002**, *41*, 2596–2599.

   doi: 10.1002/1521-3773(20020715)41:14<2596::AID-ANIE2596>3.0.CO;2-4

6) Wyszogrodzka, M.; Haag, R. *Chem. Eur. J.* **2008**, *14*, 9202–9214.

   doi: 10.1002/chem.200800892

7) Ahrendt, K. A.; Borths, C. J.; MacMillan, D. W. C. *J. Am. Chem. Soc.* **2000**, *122*, 4243–4244.

   doi: 10.1021/ja000092s

8) Sonoda, Y.; Goto, M.; Tsuzuki, S.; Tamaoki, N. *J. Phys. Chem. A* **2006**, *110,* 13379–13387.

   doi: 10.1021/jp064937j

9) Paras, N. A.; MacMillan, D. W. C. *J. Am. Chem. Soc.* **2001**, *123,* 4370–4371.

   doi: 10.1021/ja015717g

10) Riente, P.; Yadav, J.; Pericàs, M. A. *Org. Lett.* **2012**, *14*, 3668–3671.
doi: 10.1021/ol301515d
